# Comparative genomic and transcriptomic analyses of Family-1 UDP glycosyltransferase in three *Brassica* species and *Arabidopsis* indicates stress-responsive regulation

**DOI:** 10.1038/s41598-018-19535-3

**Published:** 2018-01-30

**Authors:** Hafiz Mamoon Rehman, Muhammad Amjad Nawaz, Zahid Hussain Shah, Jutta Ludwig-Müller, Gyuhwa Chung, Muhammad Qadir Ahmad, Seung Hwan Yang, Soo In Lee

**Affiliations:** 1 Department of Biotechnology, Chonnam National University, Yeosu, Chonnam 59626 Korea; 2 Department of Arid Land Agriculture, King Abdul-Aziz University, Jeddah, Saudi Arabia; 3 Institut für Botanik, Technische Universität Dresden, 01062 Dresden, Germany; 4 Department of Plant Breeding and Genetics, Bahauddin Zakariya University, Multan, 6000 Pakistan; 5 Department of Agricultural Biotechnology, National Institute of Agricultural Sciences, Jeonju, 54874 Republic of Korea

## Abstract

In plants, UGTs (UDP-glycosyltransferases) glycosylate various phytohormones and metabolites in response to biotic and abiotic stresses. Little is known about stress-responsive glycosyltransferases in plants. Therefore, it is important to understand the genomic and transcriptomic portfolio of plants with regard to biotic and abiotic stresses. Here, we identified 140, 154, and 251 putative UGTs in *Brassica rapa*, *Brassica oleracea*, and *Brassica napus*, respectively, and clustered them into 14 major phylogenetic groups (A–N). Fourteen major KEGG pathways and 24 biological processes were associated with the UGTs, highlighting them as unique modulators against environmental stimuli. Putative UGTs from *B. rapa* and *B. oleracea* showed a negative selection pressure and biased gene fractionation pattern during their evolution. Polyploidization increased the intron proportion and number of UGT-containing introns among *Brassica*. The putative UGTs were preferentially expressed in developing tissues and at the senescence stage. Differential expression of up- and down-regulated UGTs in response to phytohormone treatments, pathogen responsiveness and abiotic stresses, inferred from microarray and RNA-Seq data in *Arabidopsis* and *Brassica* broaden the glycosylation impact at the molecular level. This study identifies unique candidate UGTs for the manipulation of biotic and abiotic stress pathways in *Brassica* and *Arabidopsis*.

## Introduction

Studying the similarities and differences at the genomic and transcriptomic levels to infer the function and evolution of various biological processes is known as comparative genomics and transcriptomics. The Brassicaceae family in the plant kingdom comprises a set of diverse species of great economic, agronomic, and scientific importance, including the model plant *Arabidopsis*^1^. The six most cultivated species of the genus *Brassica* include three diploid species, namely, *B. rapa* (AA, *2n* = 20), *B. nigra (*BB, *2n* = 16), and *B. oleracea* (CC, *2n* = 18), together with three amphidiploid species, i.e., *B. juncea* (AABB, *2n* = 36), *B. napus* (AACC, *2n* = 38), and *B. carinata* (BBCC, *2n* = 34). These six *Brassica* species are related to each other and their relationship has been demonstrated by cytogenetics and hybridization studies, which confirmed that the amphidiploid species are natural hybrids of the diploids^1^. The inter-relationship of the six *Brassica* species has also been confirmed at the molecular level through genome evolution and comparative sequence analysis^2^. Genomic studies are mainly focused on the cultivated *Brassica* species, and their diploid progenitors are compared with the *Arabidopsis* genome^1^. The available genome sequences of all three *Brassica* species is a unique opportunity to study the genes family evolution from diploid to allotetraploid level.

Glycosylation is one of the final steps involved in the triterpenoid biosynthesis pathway for many plant defensive compounds, such as phenolics, glucosinolates, salicylates, and anthocyanins^3–5^. Glycosyltransferase (GT) family 1 has been found to be the largest gene family in the plant kingdom^5^. GTs can transfer sugar moieties from active sugar molecules to a variety of acceptor molecules, and are, hence, referred to as UGTs^3^. These enzymes have a 44-amino acid consensus sequence near the C-terminal, referred to as the plant secondary product glycosyltransferase (PSPG) box^6–9^. Using this PSPG motif as a search tool, 107, putative UGT genes were identified in the *Arabidopsis thaliana*, genome^10^. Recently, 147 putative UGTs have been identified in both *B. rapa* and *B. oleracea*^11^. Subsequently, putative UGT genes have been identified in other plants, including *Prunus persica* (168 genes), *Malus domestica* (254), *Vitis vinifera* (184), *Linum usitatissimum* (138), *Glycine max* (148), *Zea mays* (148), *Glycine soja* (128), and *Gossypium hirsutum* (196)^12–17^. A 40% amino acid similarity can be found among the UGTs, whereas 60% or greater similarity has been observed within subfamilies^6,18–20^. Specifically, the putative role of UGTs in fiber and seed development has been examined using RNA-seq data in cotton, flax, and soybean^12,14,16^. Based on phylogeny, UGTs can be divided into 16 distinct groups (A–P). In *Z. mays*, 17 distinct phylogenetic groups (A–Q) have been observed^15^. To date, 103 GT families have been identified in the CAZy (carbohydrate-active enzyme) database (http://www.cazy.org/), which is a comprehensive resource that specializes in organizing carbohydrate-active enzymes^21,22^. GTs comprise almost 40% of the enzymes present on the CAZy website^21^.

UGTs respond to a variety of plant stresses by conjugating with various phytohormones and other metabolites by attaching the activated sugars. The role of UGTs in response to biotic stresses has been well characterized, even though their precise contribution still remains unclear^23^. For example, in tomato (*UGT73B3,* and *UGT73B5*) and *Arabidopsis* (*AtSGT1*, and *UGT76B1*), genes have been characterized in response to *Pseudomonas syringae* infection, which could possibly play a role in the crosstalk of salicylic acid (SA) and jasmonic acid (JA)^24–26^. *UGT74F1* mutants exhibit lower levels of salicylic acid (SA) and lower levels of resistance to bacterial infection from *P. syringae*. In contrast, *UGT74F2* mutants have higher levels of SA and higher levels of resistance to *P. syringae*. Similarly, overexpression of *UGT74F2* (also annotated as *AtSGT1*) results in lower levels of SA and an increased susceptibility to *P. syringae*^26–28^. According to the results of a recent study, the expression of a *BSMT* gene in *B. oleracea* appears to be involved in glycosylation rather than methyl jasmonate (MeJA) biosynthesis during *Plasmodiophora brassicae* infection^29^. In tobacco, the glycosylation of phenylpropanoids played a significant role during tobacco mosaic virus (TMV) infection and the down-regulation of a tobacco glycosyltransferase gene (*TOGT1*) led to reduced TMV resistance by storing the aglycone in the form of scopolin (a glycoconjugated coumarin)^23,30^. Overexpression of the TOGT genes also increased resistance against the potato virus Y (PVY) in tobacco^31^. Accumulation of sinapate esters, coniferin, and glycosylated pinoresinol and lariciresinol lignans was observed when *Arabidopsis* leaves were infected by the soil-borne ascomycote, *Verticillium longisporum*^23,32^. Transgenic plants that overexpressed *UGT72E2* were less susceptible to this fungi, owing to the accumulation of coniferin^23^. Plant species can also display glycosylation-related cultivar- and pathogen-dependent responses to biotic stresses^23^. An upregulation of cytochrome P450 (*CYP709C1*) and UGT-encoding genes in wheat was observed during the infection by *Fusarium graminearum*, compared with *Magnaporthe grisea*. The cultivar resistance to *F. graminearum* led to a stronger expression in incompatible (resistant) interactions with the Chinese spring wheat cultivar, Sumai 3^23,33^. The constitutive overexpression of the (*UGT73C5*) gene in *Arabidopsis* led to an enhanced tolerance against the deoxynivalenol mycotoxin produced by *Fusarium*, by glycosylating it into DOGT1^34^. These studies underline the importance of some UGT genes implicated in pathogen response and redox status, during pathogen infection. To date, *UGT84F1* in *B. napus* and *BrUGT* in *B. rapa* has been functionally characterized for inhibition of the biosynthesis of sinapine and abiotic stress tolerance, respectively^35^.

The biological role of the putative UGTs in response to abiotic stresses is largely unknown. For example, in *Arabidopsis*, *UGT85U1/2* and *UGT85V1* were found to be involved in salt and oxidative stress tolerance by changing the composition of several indole derivatives^36^. In tobacco, ectopic expression of *UGT85A5* resulted in enhanced salt stress tolerance in transgenic plants^37^. Furthermore, in *Arabidopsis*, ectopic over-expression of *UGT74E2* increased the tolerance to salinity and drought stress, and reduced the water loss in plants^38^, while 11 UGTs were up-regulated in response to H_2_O_2_ stress in catalase-deficient plants^39^. Recently, *UGT85C2*, *UGT74G1*, and *UGT76G1* were shown to be downregulated under polyethylene glycol-induced drought stress in *Stevia rebaudiana*^40^. The role of abscisic acid (ABA) in mediating drought stress has been extensively studied and several UGTs have been functionally characterized for ABA-glucose ester formation^41–44^. ABA glycoconjugate genes from various plants have been characterized, including *UGT71A33*, *UGT71A34a/b* and *UGT71A35* (strawberry), *UGT87A2*, *UGT71B7*, *UGT71B8*, *UGT71B6*, and *UGT71C5* (*Arabidopsis*), *ABAGT* (*Phaseolus vulgaris*), *UGT73C14* (*Gossypium hirsutum*), and *ABAGT* (*Vigna angularis*)^41–45^.

An extensive research interest have attracted the plant UGTs in response to physiological function as well as their potential biotechnological applications^46^. It will be essential to integrate data from *in vitro* and *in vivo* studies across crops to obtain a complete picture of the potential biological and molecular roles of UGTs in plants^46^. Here, we employed bioinformatics techniques to identify 545 putative UGTs in three *Brassica* species, and then subjected these to genomic and transcriptomic analyses. To the best of our knowledge, the present study identified UGT genes at a genome-wide scale in *B. napus*, *B. oleracea*, and *B. rapa*, and compare these with *Arabidopsis*; our findings provide insights into the evolutionary history of UGT genes and their putative roles in development, as well as the response to biotic and abiotic stresses.

## Results

### Identified Putative UGTs in Brassica

We identified 545 putative UGTs in three *Brassica* species, with a PSPG (Plant Secondary Product Glycosyltransferase) motif at their C-terminal from the projected proteomes (Supplementary Table S1), having a sequence similarity of 40% to 98% with the *Arabidopsis* UGTs (Supplementary Fig. S1). Out of these 545 putative UGTs, 140 belong to *B. rapa*, 154 to *B. oleracea*, and 251 to *B. napus*. The PHMMR tool used for the identification of UGTs produced consistent results in terms of the UDPGT domain with PFAM00201. The average peptide lengths and molecular weights of the putative UGTs were found to be almost similar across the species, with an average length and weight of 438 aa and 49 kDa in *B. napus*, 457 aa and 51 kDa in *B. oleracea*, and 454 aa and 51 kDa in *B. rapa* (Supplementary Table S1). In *B. rapa*, *Bra034848.1* was the gene with a maximum length of 1183 aa among all three *Brassica* species, and it had a PSPG motif. All the UGT sequences started with a methionine and were full-length sequences. The percentage of GC content was also found to be similar among all *Brassica* species putative UGTs, with a range of 44.36% to 45% (Supplementary Table S1). As per the calculated amino acid composition for the putative UGTs in all three *Brassica* species, the amino acids, leucine, valine, glycine and aspartate were predominant across all the UGT proteins (Table S1). Cysteine and tyrosine were found minimally.

### Phylogenetic Analysis and Comparison

**Figure 1:**
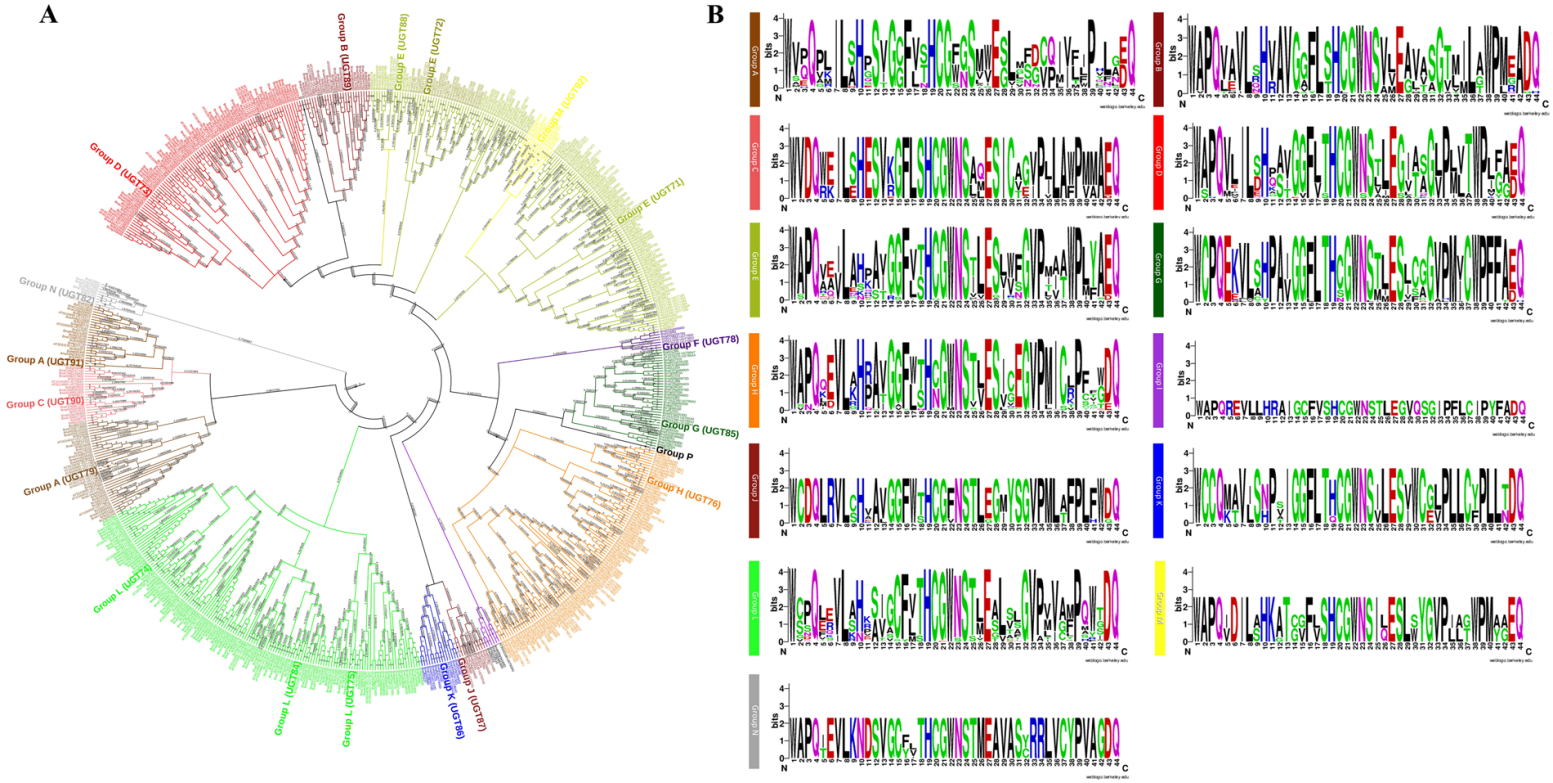
(**A**) The evolutionary history was inferred using the Neighbor-Joining method. The optimal tree with the sum of branch length = 4816.24407500 is shown. The tree is drawn to scale, with branch lengths in the same units as those of the evolutionary distances used to infer the phylogenetic tree. The evolutionary distances were computed using the number of differences method and are in the units of the number of amino acid differences per sequence. The analysis involved 567 amino acid sequences. All positions with less than 80% site coverage were eliminated. That is, fewer than 20% alignment gaps, missing data, and ambiguous bases were allowed at any position. There were a total of 171 positions in the final dataset. Evolutionary analyses were conducted in MEGA7. (**B**) PSPG motif of each phylogenetic group is shown.

The final alignment file contained the aligned positions with two highly conserved sites, 490 variables, and 17 singleton sites. The constructed phylogenetic tree suggested a classification into 14 major groups (A–N), as seen in all higher plants such as maize, cotton, peach, and apple (Fig. 1A)^12,14,15,17^. Two newly discovered groups (O and P) were absent in all three *Brassica* species as well as *Arabidopsis*, but are present in *Z. mays*^15^. The PSPG motif was found to vary in each phylogenetic group, but was similar across all three *Brassica* species and *Arabidopsis* (Supplementary Table S1 and Fig. 1B). The histidine at positions 10 and 19 in the PSPG motif was the most conserved site across all three *Brassica* species (Fig. 1B).

**Figure 2:**
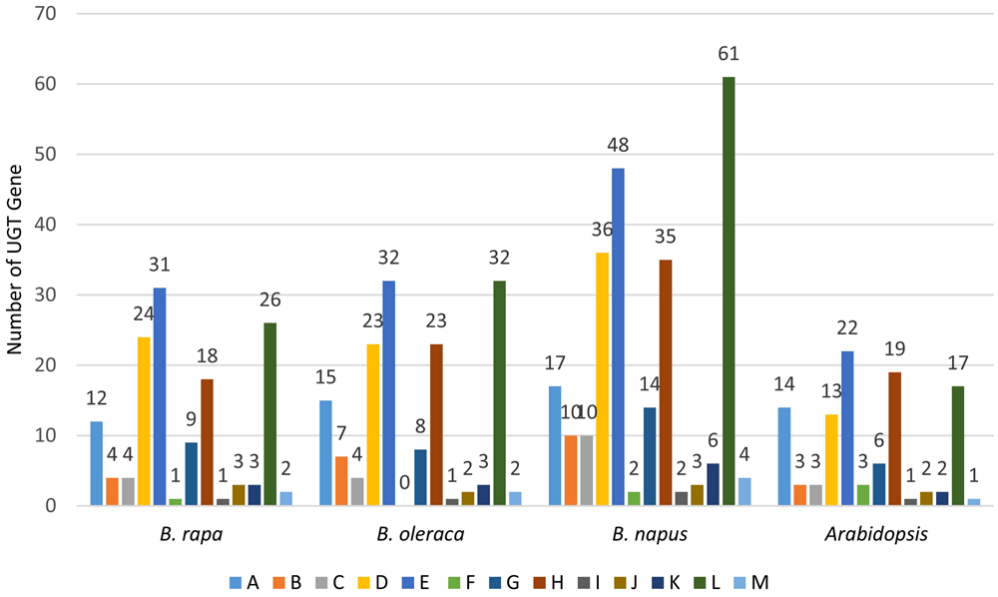
Comparison of phylogenetic group of UGTs among all three *Brassica* species and *Arabidopsis*. Each phylogenetic group is representing the number of UGT genes in that group.

Across the *Brassica* and *Arabidopsis* species, groups D, E, H, and L had the largest numbers of UGTs (Fig. 2). Phylogenetic groups E and L in *B. napus* were the most expanded ones among all the compared groups, with 48 and 61 genes, respectively (Fig. 2). Groups I, J, K, M, and N were found to be conserved among all three *Brassica* species (Fig. 2). *B. napus* had the maximum number of putative UGTs in each group, while *B. rapa* and *B. napus* had almost the similar number of genes in their phylogenetic groups. In *B. oleracea*, group F was found to be absent.

### KEGG Pathway Mapping and Go Annotation

**Figure 3:**
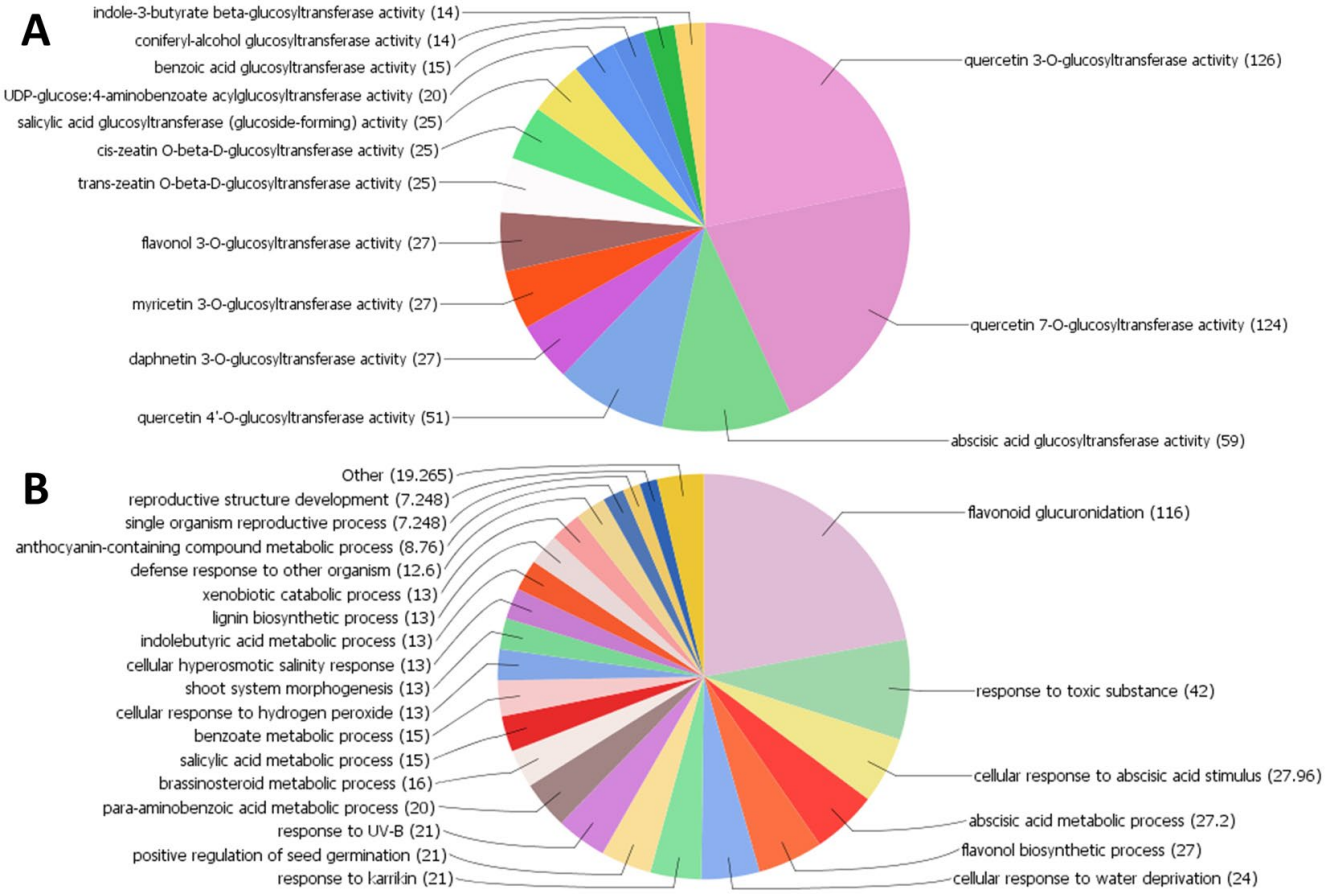
Go ontology analysis of *Brassica* UGTs. (**A**) Molecular function analysis of putative UGTs. (**B**) Biological process involvement of putative UGTs.

The Go ontology analysis of all the identified putative UGTs from all three-brassica species associated them with 13 molecular function and 24 biological processes shown in (Fig. 3A and B). In general, fourteen major KEGG pathways (steroid hormone biosynthesis, metabolism of xenobiotics by cytochrome P450, zeatin biosynthesis, phenylpropanoid biosynthesis, pentose and glucuronate interconversions, tryptophan metabolism, drug metabolism - other enzymes, flavone and flavanol biosynthesis, ascorbate and aldarate metabolism, glucosinolate biosynthesis, drug metabolism - cytochrome P450, retinol metabolism, porphyrin and chlorophyll metabolism, and anthocyanin biosynthesis) were found to be linked with the putative UGTs (Supplementary Table S2). Phylogenetic group wise KEGG pathway mapping was also investigated and listed in (Supplementary Table S2). Each group UGTs were found to be involved for specific KEGG pathways. The putative UGTs involvement in various biological processes and their molecular functions is presented in group wise (Supplementary Fig. S2). Overall most of the putative UGTs were found to be involved in quercetin 3-O- and 7-O glucosyltransferases, hormonal glycosylation and various flavonoid glucuronidation and their biosynthesis.

### Chromosomal distribution, Duplication, and Divergence

**Figure 4:**
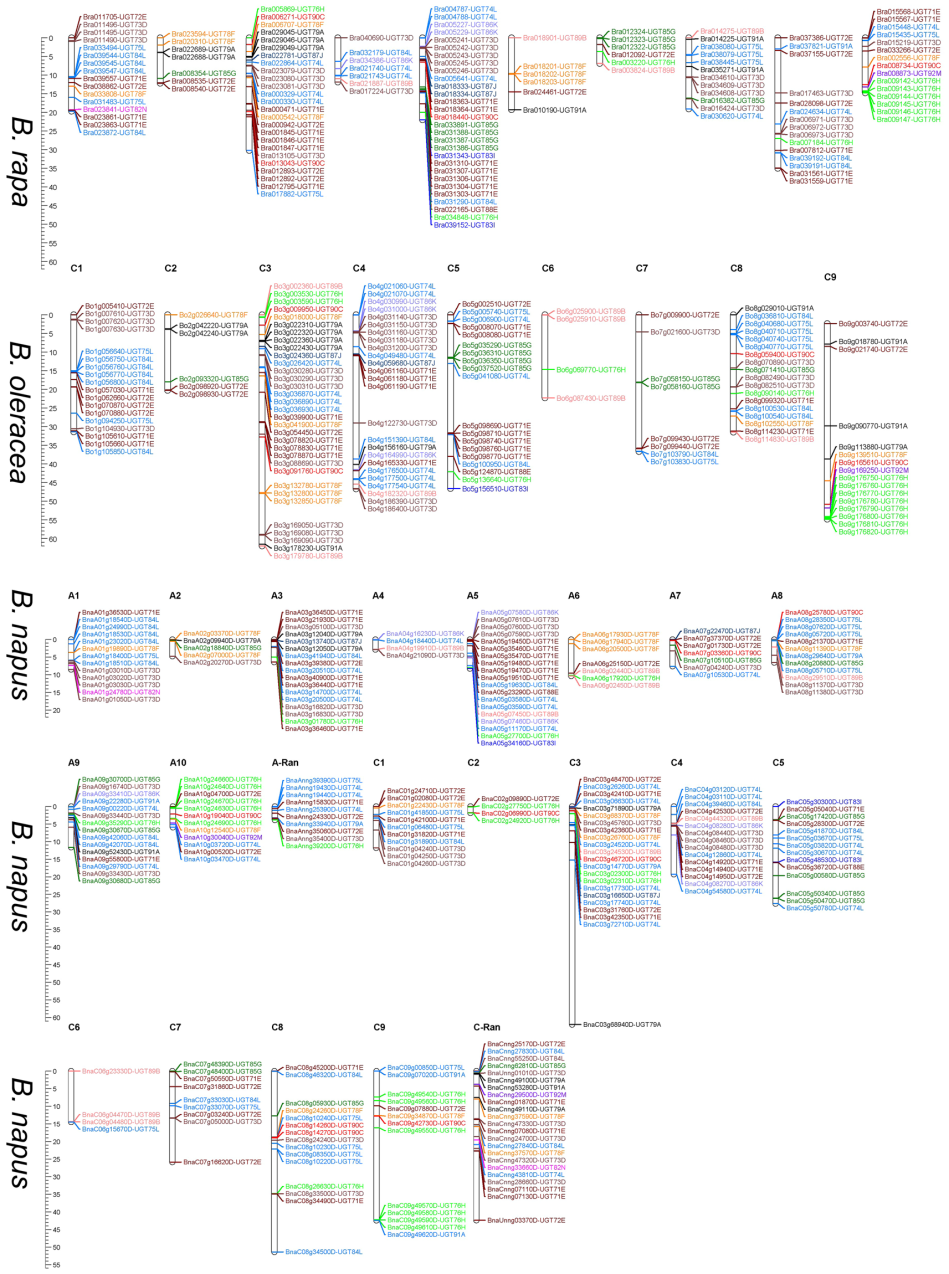
Chromosomal distribution of putative UGTs in all three *Brassica* species. Individual UGTs position are shown in Mbs alongside the chromosome scale. Different colors are representing each phylogenetic group of UGTs.

All the chromosomes in all three *Brassica* species contained UGTs (Fig. 4), although the numbers varied among them. In *B. napus*, some genes were found without any chromosome number and were included in the random category (Fig. 4). A comparison between *B. rapa* and *B. napus* AA chromosomes showed that the putative UGTs of *B. napus* followed the exact pattern of chromosome involvement as its progenitor *B. rapa* (Fig. 4). The same was observed for CC genome from *B. oleracea* and *B. napus*.

The maximum clustering of the putative UGTs in all three *Brassica* species was observed in phylogenetic groups E, L, and H (Fig. 4). Cluster size was found to be variable for each chromosome in all three *Brassica* species (Fig. 4).

We found 28 clusters of tandemly duplicated UGTs in *B. rapa* (Supplementary Table S3, Supplementary Fig. S4). The maximum clusters consisted of two to four UGT genes. Some clusters had five to seven UGT genes, for example Cluster_17 and Cluster_13 (Supplementary Fig. S4). For *B. rapa*, almost all the clusters belonging to the same phylogenetic groups, except some clusters, such as Cluster_171, which had a gene from phylogenetic groups A and D (Supplementary Table S3). We also found 17 clusters of tandemly duplicated UGTs in *B. oleracea* (Supplementary Fig. S4, Supplementary Table S3). The cluster size in *B. oleracea* was 2 to 4 genes per cluster. We also found that some cluster genes belonged to two different phylogenetic groups, such as the genes in Cluster_1498, Cluster_453, and Cluster_1455 in *B. oleracea*.

We found that 25 duplicated pairs of putative UGTs diverged between *B. rapa* (AA) and *B. oleracea* (CC) genomes at the time of divergence from their progenitor (Table 1). Almost all the duplicated UGT gene pairs belonged to those genes that had undergone a genes that had undergone a whole-genome duplication (WGD) or segmental duplication, and all the pairs had a Ka/Ks ratio of less than 1, indicating the purifying selection acting on these genes. The calculation of the divergence time of the duplicated UGTs spanned from 1.41 to 21.07 million years ago (MYA) (Table 1). Duplicated gene pairs 2,5,11, 17, 18, and 21 diverged around 13.65 to 21.07 MYA, while fourteen gene pairs (1, 3, 6, 7, 8, 9, 10, 12, 13, 14, 15, 16, 19, 20, 22, 24, and 25) diverged around 1 to 5 MYA. Only two UGT genes pairs diverged around 5 to 9 MYA.

**Table 1:** Calculation of Ka/Ks and the divergence time of the duplicated UGT gene pairs in *B. rapa* (AA) and *B. oleracea* (CC) genomes.

Duplicated Gene Pairs	Ka	Ks	Ka/Ks	Duplication type	Purifying Selection	Time (MYA)
1. *Bo1g005410-Brara.A00194*	0.0306	0.133	0.22	WGD/Segmental	Yes	4.45
2. *Bo4g122730-Brara.I03603*	0.0802	0.370	0.21	WGD/Segmental	Yes	12.36
3. *Bo4g122730- Brara.D00540*	0.0086	0.424	0.23	WGD/Segmental	Yes	1.41
4. *Bo8g082490- Brara.I03603*	0.0562	0.178	0.31	WGD/Segmental	Yes	5.94
5. *Bo8g082490- Brara.D00540*	0.0753	0.419	0.17	WGD/Segmental	Yes	13.98
6. *Bo8g082510- Brara.I03606*	0.0178	0.091	0.19	WGD/Segmental	Yes	3.06
7. *Bo3g041900- Brara.C02459*	0.0174	0.080	0.21	WGD/Segmental	Yes	2.69
8. *Bo5g156510- Brara.E03602*	0.0181	0.06	0.30	WGD/Segmental	Yes	2
9. *Bo4g030990- Brara.E00800*	0.0145	0.088	0.16	WGD/Segmental	Yes	2.96
10. *Bo4g164990- Brara.D01677*	0.0096	0.106	0.09	WGD/Segmental	Yes	3.53
11. *Bo4g059680- Brara.C01536*	0.164	0.63	0.26	WGD/Segmental	Yes	21.07
12. *Bo5g124870- Brara.E02501*	0.016	0.09	0.17	WGD/Segmental	Yes	3.26
13. *Bo3g179780- Brara.H00222*	0.0184	0.12	0.15	WGD/Segmental	Yes	4.14
14. *Bo6g025900- Brara.F00261*	0.048	0.012	4	WGD/Segmental	No	4.05
15. *Bo6g087430- Brara.G02296*	0.025	0.09	0.27	WGD/Segmental	Yes	3.3
16. *Bo3g009950- Brara.C00639*	0.019	0.061	0.31	WGD/Segmental	Yes	2.03
17. *Bo3g009950- Brara.J01993*	0.100	0.42	0.23	WGD/Segmental	Yes	14.29
18. *Bo3g091760- Brara.G00317*	0.102	0.40	0.25	WGD/Segmental	Yes	13.65
19. *Bo3g091760- Brara.C04165*	0.020	0.98	0.02	WGD/Segmental	Yes	3.27
20. *Bo8g059400- Brara.H02763*	0.015	0.07	0.21	WGD/Segmental	Yes	2.65
21. *Bo9g165610- Brara.C00639*	0.10	0.43	0.23	WGD/Segmental	Yes	14.48
22. *Bo9g165610- Brara.J01993*	0.017	0.07	0.24	WGD/Segmental	Yes	2.63
23. *Bo9g018780- Brara.I00824*	0.10	0.19	0.52	WGD/Segmental	Yes	6.64
24. *Bo9g090770- Brara.I02539*	0.017	0.05	0.34	WGD/Segmental	Yes	1.67
25. *Bo9g169250- Brara.J02137*	0.01	0.081	0.12	WGD/Segmental	Yes	2.71

### Gene synteny and subgenomic fractionation of the putative UGTs in *Brassica*

**Figure 5:**
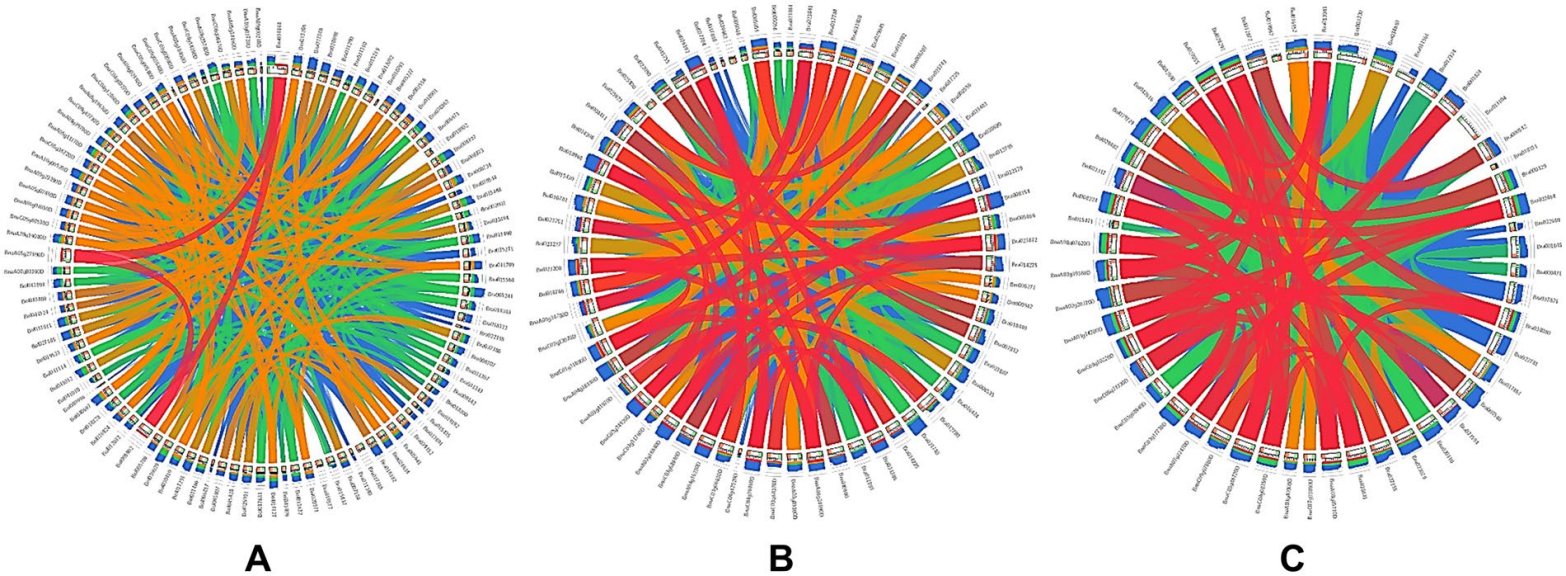
Subgenome fractionation of putative UGTs among *Brassica*. (**A**) representing UGTs in LF subgenome (**B**) representing MF1 subgenome UGTs (**C**) representing MF2 subgenome UGTs. Ribbons represent the local alignments BLAST in four semi-transparent colors, blue, green, orange and red, representing the four quartiles up to the maximum score - i.e. a local alignment with a score of 80% of the maximum score is red, while one with 20% of the maximum score is blue.

*B. rapa* showed paleo hexaploidy, with three subgenomes that share the same diploid ancestor as that of the model species *A. thaliana*^47^. Here, we provide *B. rapa*-orthologous syntenic genes in *B. napus*, *B. oleracea*, and the model plant *Arabidopsis*. We found, 100 UGT genes of *B. rapa* were having the syntenic genes in all three-compared species including *Arabidopsis* (Supplementary Table S3). Overall, All the syntenic genes belonged to tPCK (the ancestral genome of *Brassica* species) chromosomes 1 to 7 and the following syntenic blocks: A, B, C, E, F, H, I, J, M, N, O, R, T, U, Wb, and x (Supplementary Table S3). The maximum number of syntenic UGT genes was retained in the fractioned subgenome (LF) of *B. rapa*, *B. oleracea*, and *B. napus* (Supplementary Table S3). The minimum number of orthologs was contained in the more fractioned subgenome (MF2) of all three *Brassica* species. Each phylogenetic group of the putative UGTs belongs to different syntenic blocks, for example, in group E, different syntenic blocks (A, F, T, J, O, X, N) were detected (Supplementary Table S3). Similarity in the syntenic sequences of the putative UGTs among LF, medium fractionated subgenomes (MF1), and MF2 subgenomes are shown in (Fig. 5).

### Exon/Intron organization of the putative UGTs in *Brassica*

Of the 545 *UGT*s, 219 possess no introns, whereas 326 had introns (Supplementary Table S4). Out of the 326 intron-containing UGTs, 71, 80, and 175 genes were found in *B. rapa*, *B. oleracea*, and *B. napus*, respectively. A ratio of 1.35, 1.25, and 0.7 introns per intron-containing UGT was observed in *B. rapa*, *B. oleracea*, and *B. napus*, respectively (Supplementary Table S4). Except *Bra034848*, which had 11 introns, almost all the remaining UGTs had 1–4 introns per gene. In each phylogenetic group, the intron numbers were found to vary among the three *Brassica* species. The maximum number of introns was found in E, D, L, and H, whereas the minimum number of introns was found in B, C, F, I, M, and N groups, in all three *Brassica* species (Supplementary Table S4). Intron phases in some phylogenetic groups were also found to be conserved; for example, all the introns in group G UTGs were found in phase 1 in *B. rapa* and *B. oleracea* (Supplementary Table S4). Intron size varied in each phylogenetic group across the three *Brassica* species (Supplementary Table S4). Interestingly, *B. rapa* and *B. oleracea* followed the same pattern of introns in each phylogenetic group, for example, the group A UGTs did not have any intron in either species.

After mapping the introns to the amino acid sequence alignments, at least 10 independent intron insertion events can be observed in intron-containing UGTs, following an established method^12,17^. These insertion events can be serially numbered I-1 to I-10, according to their positions. Intron insertion in phase 1 between 150–200 aligned amino acids was found to be highly conserved across the three *Brassica* species and *Arabidopsis*^10^ (Supplementary Fig. S4). For group G, in the UGTs from all three *Brassica* species, a highly conserved intron-insertion region among the amino acids could be observed. A deletion of the conserved intron-insertion region was observed in group E UGTs for all three *Brassica* species (Supplementary Fig. S4).

### Transcriptome analysis of tissue-specific expression

The RNA-seq data (GSE43245) from *B. rapa* tissues showed that the UGTs showed a variable expression in all the seven investigated tissues (callus, flower, silique, leaf, root, and stem) (Supplementary Fig. S5A). The maximum expression of these UGTs was observed in callus, flower, root, and silique tissues. The same results for UGT gene expression were observed in the 127 investigated anatomical parts in *Arabidopsis* (Supplementary Fig. S5B), with the maximum expression observed in silique (endosperm and testa) and root (root protoplast, root cortex, and root stele cells). Some UGT genes in *Arabidopsis*, such as *UGT76F2*, *UGT85A1*, *UGT85A5*, *UGT87A2*, and *UGT89A2* were preferentially expressed in the leaf epidermis and guard cells (Supplementary Fig. S5B). *UGT72B7* genes in *Arabidopsis* were maximally expressed in the shoot vascular and phloem companion cells. *UGT76E3* and *UGT84B1* were preferentially expressed in the seed endosperm in *Arabidopsis*. Overall, each UGT gene showed a unique expression pattern, and therefore, we cannot link the expression patterns with the phylogenetic groups (Fig. S4B). The investigated expression of the *Arabidopsis* UGTs across 10 developmental stages (germinated seed, seedlings, young rosette, developed rosette, bolting, young flower, developed flower, Flower/silique, silique, and senescence) suggested that the UGTs had the maximum expression at the senescence stage (Supplementary Fig. S5C).

### Transcriptome analysis of *Plasmodiophora brassicae*

Glycosylation of fungal-responsive pathway proteins was evaluated using RNA-seq data in *Arabidopsis* and *B. rapa*. RNA- seq data at 24 hai and 48 hai stages, in response to *P. brassicae*, were investigated in *Arabidopsis*^48^. The maximum number of UGT genes were found to be downregulated upon early and late infection by *P. brassicae*, in both *Arabidopsis* and *B. rapa* (Supplementary Table S5, Supplementary Fig. S6A). Only 9 UGT genes (*UGT71B3P*, *UGT71C1*, *UGT72D1*, *UGT72E1*, *UGT72E2*, *UGT74B1*, *UGT74C1*, and *UGT76F2*) were found to be upregulated, based on the Reads Per Kilobase of transcript per Million mapped reads (RPKM) values for infection with *P. brassicae*. Some genes were up- and downregulated simultaneously, but at different stages of infection, for example the UGT74E2 gene was found to be upregulated after 48 hai, whereas downregulated at 24 hai, compared to the control. UGT71C1, UGT72E1, and UGT72E2 showed the maximum TPM values against *P. brassicae* infection. The UGT79B5 gene in *Arabidopsis* was maximally downregulated, with RPKM reduction from 229 to 106, against the clubroot pathogen.

Based on the RPKM values, the maximum number of downregulated UGTs against *P. brassicae* was observed in the clubroot-resistant line compared to the susceptible line of *B. rapa*^49^ (Supplementary Table S5, Supplementary Fig. S6B). All the downregulated UGTs had low RPKM values against early and late infectious stages of *P. brassicae*. Twenty-one UGTs were found to be upregulated against the clubroot disease. Interestingly, UGTs from group M were found to be upregulated in both *Arabidopsis* and *B. rapa* against clubroot.

### Differentially expressed UGT genes for Phytohormone Glycosylation

**Figure 6:**
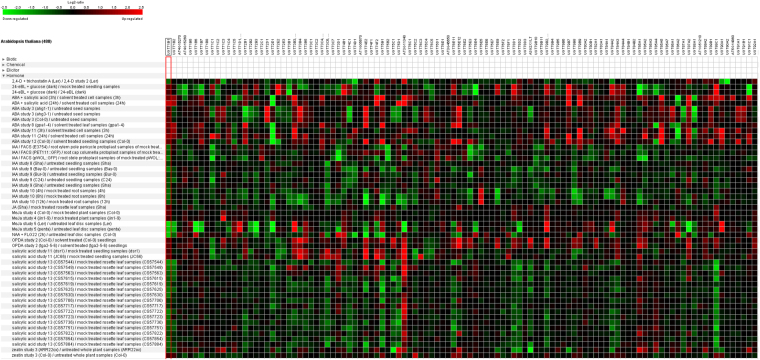
Differential expression of up and downregulated UGTs of *Arabidopsis* in response to different hormonal treatments (2,4-D + trichostatin, ABA + SA, IAA, MeJA, SA, and zeatin). Green color is showing down regulated UGTs and Red color is showing up regulated UGTs.

Diversity in the putative UGTs to glycosylate phytohormones was evaluated using microarray analysis in *Arabidopsis*, and further confirmed in *B. rapa* for MeJA-treated RNA-seq data. All the 118 UGTs were differentially expressed against 2,4-D + trichostatin, ABA + SA, indole acetic acid (IAA), MeJA, SA, and zeatin (Fig. 6). The fold change and p-values are provided in Fig. 6. *UGT71C3*, *UGT74D1*, and *UGT90A2* genes were downregulated in the hypocotyl segments treated with 2,4-D and trichostatin (a histone deacetylase inhibitor) (Fig. 6). *UGT72E2*, *UGT74B1*, and *UGT79B7* were found to be upregulated in response to 2,4-epibrassionolide and in glucose-treated seedlings (Fig. 6). A differential expression of *UGT71B7*, *UGT85A5*, and *UGT87A2* was observed in the cell suspension cultures under 25 uM ABA and 300 uM SA after 3 h of treatment. Interestingly, *UGT72E1* was downregulated in the leaf samples treated with 50 mM ABA for 3 h, and cell samples treated with 25 uM ABA after 3 and 24 h. An upregulation of *UGT72D1*, *UGT73B4*, and *UGT76E12* was observed in the seeds of ABA-hypersensitive mutant (ahg1-1), germinated on a medium containing 0.5 uM ABA. A downregulation of *UGT71C1* and *UGT73D1* was observed in the root xylem pole pericycle protoplast samples transferred to 5uM IAA solution for 3 h. *UG74E2* and *UGT75B1* were maximally downregulated in the root samples of Col-0 grown for 6 days under continuous light and then transferred to 1 uM IAA for 8 and 12 h. *UGT71B7*, *UGT72B3*, *UGT72E1*, *AT3G11340*, *UGT78D2*, and *UGT87A2* were the most downregulated UGT genes in the leaf disc samples of Ler and Penta plants grown under short-day light conditions and sprayed with 10 uM MeJA. *UGT71D1*, *UGT73B3*, *UGT79B2*, and *UGT90A1* genes were upregulated under 10 uM MeJA-treated leaf disk samples from Ler and Penta plants of *Arabidopsis*. *UGT74D1* and *UGT74E2* were found to be downregulated in the leaf discs samples treated with 10 uM naphthalene acetic acid NAA (Fig. 6). *UGT73E4* and *UGT76E12* were upregulated in response to Col-0 plant samples treated with 75 uM 12-oxo-phytodienoic acid (OPDA) for 4 h. A consistent upregulation of *UGT76B1* and down regulation of *UGT89C1* was observed in rosette leaf samples taken from plants of 16 different genetic background, treated with 0.3 mM SA. *UGT75C1* and *UGT78D3* were the only downregulated UGT genes in 21-day old whole plants, which overexpressed the ARR22 (Col-0 background) and were treated with 20 uM trans-zeatin for 3 h (Fig. 6). To compare this data, we found only the RNA- seq (GSM1243356) dataset of *B. rapa* leaves treated with 0.2 mM MeJA for ZSN5 cultivar. We found 50 upregulated and 39 downregulated UGT genes in response to MeJA exposure (Supplementary Table S6, Supplementary Fig. S6). *Bra031290*, *Bra023872*, *Bra023594*, *Bra009143*, and *Bra005641* were the most down regulated UGT genes based on the RPKM values under MeJA treatment. *Bra039547*, *Bra015435*, and *Bra012892* were highly upregulated in response to MeJA (Supplementary Table S6, Supplementary Fig. S7).

### Differentially expressed *Arabidopsis* UGT genes under Biotic and Abiotic stresses

**Figure 7:**
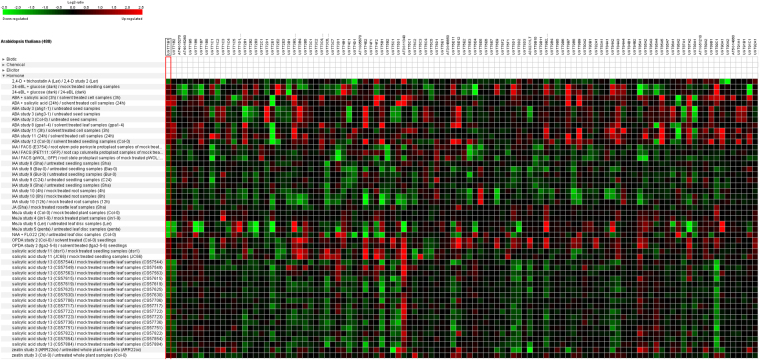
Differential expression of up and downregulated UGTs of *Arabidopsis* in response to different pathogen infestations (*A. brassiciola, B. graminis, E. coli, G. cichoracearum, G. orontii, H. arabidopsidis, P. cuccumerina, P. syringae, R. solani*, and *X. campestris*). Green color is showing down regulated UGTs and red color is showing up regulated UGTs.

Overall, the maximum number of UGT genes in *Arabidopsis* was found to be upregulated in response to pathogens such as *Alternaria brassiciola, Blumeria graminis, Escherichia coli, G. cichoracearum, Golovinomyces orontii, Hyaloperonospora arabidopsidis, Plectospherella cuccumerina, P. syringae, Rhizoctonia solani*, *and Xanthomonas campestris* (Fig. 7). Interestingly, *UGT73B3, UGT73B4*, and *UG73B5* were differentially upregulated in response to all the above-mentioned pathogens. *UGT71C3*, *UGT74E2*, and *UGT85A1* were highly upregulated in rosette leaf samples treated with the pathogens, *A. brassicicola* and *B. graminis*. A very low level of gene regulation of UGTs was observed against *E. coli*, *H. arabidopsidis*, and *R. solani* in *Arabidopsis*. The *UGT89C1* gene was highly downregulated in response to *G. cichoracearum-*treated whole rosette leaf samples after 18 h and 36 h in cultivars of Col-0 and edr1 genetic background (Fig. 7). *UGT73B3* and *UGT89C1* were upregulated against *G. orontii* infection in the rosette leaf samples in plants of Col-0 and eds16-1 genetic background. *UGT73B3*, *UGT76C1*, *UGT85A1*, and *UGT87A1* responded positively in response to *P. cucumerina* treatment in rosette leaf samples of plants with agb1-1 genetic background. An upregulation of *UGT73D1* was observed against *P. syringae* pv. phaseolicola in the leaves of Col-0 plants. The maximum number of UGT genes was found to be upregulated in response to *P. syringae* treatment in plants (Fig. 7). Again, *UGT73B3* responded positively in terms of gene expression against *X. campestris* treatment in the leaves of plants with Ws-4 genetic background.

**Figure 8:**
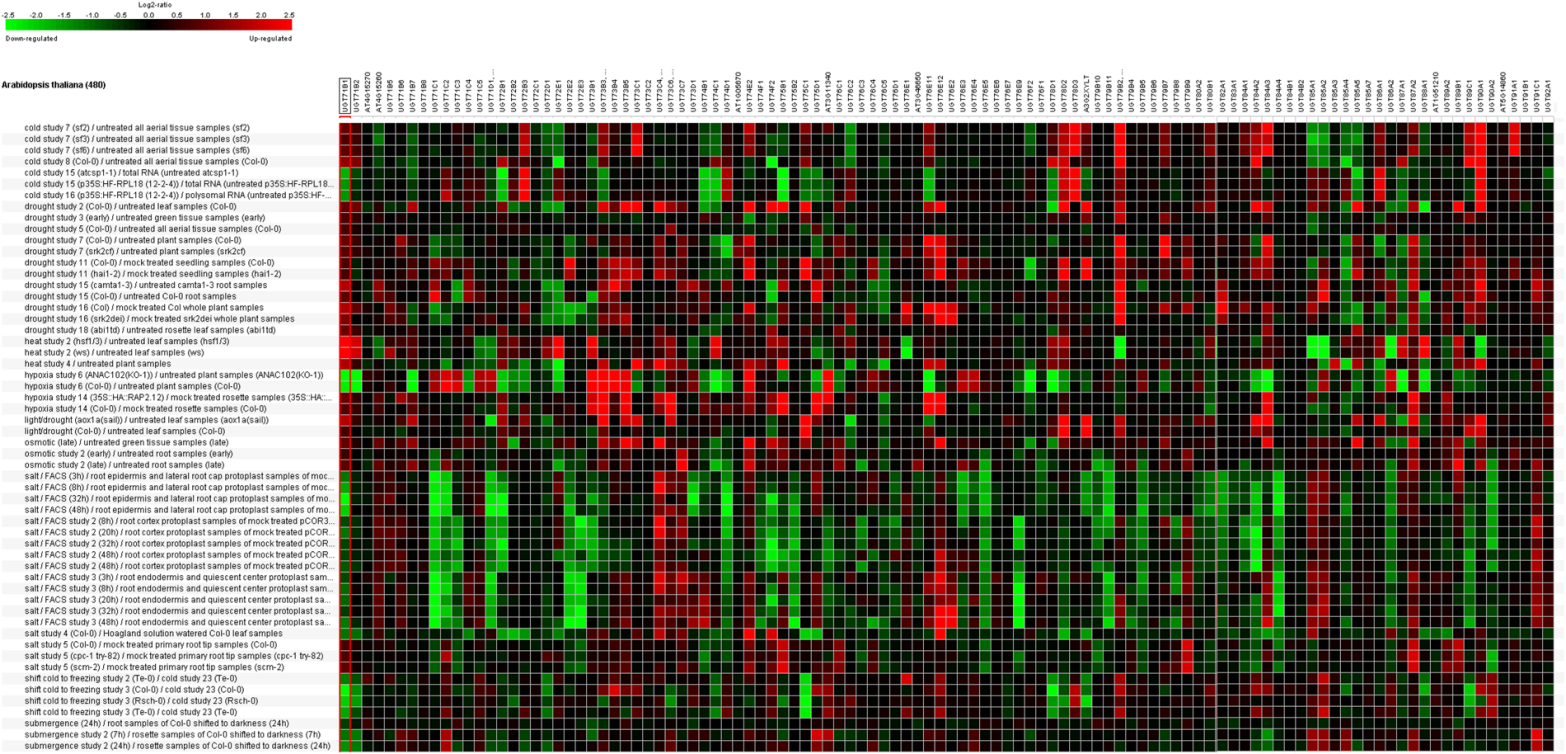
Differential expression of up and downregulated UGTs of *Arabidopsis* in response to different abiotic stresses (cold, drought, heat, hypoxia, osmotic, salt, and submergence conditions). Green color is showing down regulated UGTs and red color is showing up regulated UGTs.

A diverse range of differentially expressed UGT genes of *Arabidopsis* was observed against cold, drought, heat, hypoxia, osmotic, salt, and submergence conditions (Fig. 8). *UGT73C1*, *UGT78D3*, *UGT79B2*, *UGT84A3*, and *UGT90A1* were dominantly expressed in the aerial tissue samples of sf2 plants, grown at 21 °C under 24 h illumination and then exposed to cold (4 °C) for 24 h. A downregulation of *UGT72E1* and *UGT74F2* was observed in the aerial tissue samples of Col-0 plants, grown at 20 °C under 8 h light/16 h dark for 28 days and then exposed to cold (4 °C) for 10 days. *UGT72B1* and *UGT75B1* were downregulated, whereas *UGT78D3* and *UGT90A1* were upregulated in rosettes of atcsp1-1 (a knockdown mutant) plants maintained at 4 °C. In drought, *UGT73B3*, *UGT73B5*, *UGT73C1*, *UGT74E2*, and *UGT79B1* were highly uregulated in leaf samples from wild type (Col 0) plants, which had not been watered for 7 days. *UGT79B2*, *AT4G31780*, *UGT85A1*, *UGT85A2*, *UGT86A2*, and* UGT90A1* were downregulated in hsf1: hsf3 (mutant plants significantly impaired with respect to the induction of HS gene expression) leaf samples after 1-h heat treatment (37 °C). In response to hypoxia, *UGT71B1*, *UGT71B2*, *UGT71B7*, *UGT74D1*, *UGT76E1*, *UGT76F2*, *UGT78D1*, *UGT87A1*, and *UGT88A1* were highly downregulated in ANAC102(KO-1) (mutant) plants exposed to low oxygen treatment for 4 h in a dark chamber. *UGT73B3*, *UGT73B5*, and *UGT73B6* were found to be upregulated in response to the low oxygen treatment. A very low level of regulation of UGT genes was observed in response to 300 mM mannitol-treated leaf samples of col-0 plants. The maximum number of UGT genes was found to be highly downregulated, such as *UGT71B1*, *UGT71C1*, *UGT71C2*, *UGT71B1*, *UGT71D1*, *UGT72B2*, *UGT74D1*, *UGT74F2*, *UGT76E9*, *UGT84A2*, and *UGT84A4*, in root epidermis and lateral root cap protoplast samples treated for 3 h (Fig. 8). A downregulation of *UGT75C1* and *UGT78D1* was observed in the leaf samples shifted from cold to freezing conditions.

**Figure 9:**
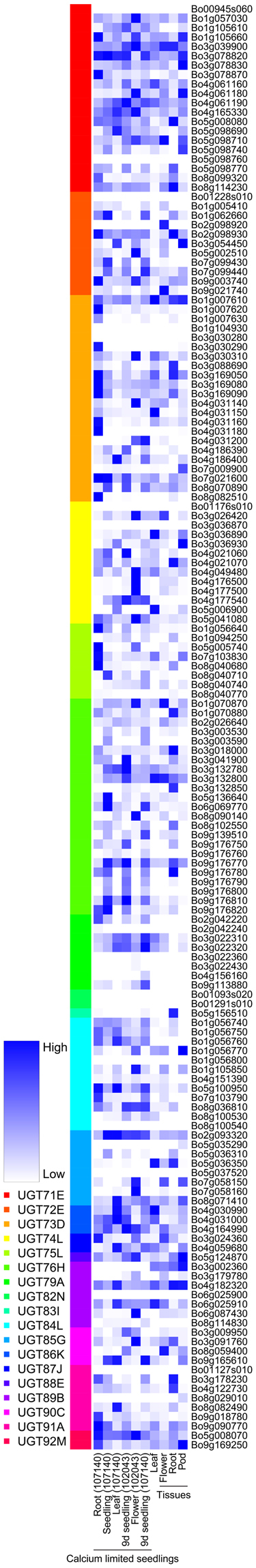
Differential expression of up and down regulated UGTs of *B. oleracea* in response to leaf, flower, pod, root, and calcium limited seedling tissues i.e. root, leaf and flower. Dark blue color is showing up-regulated UGTs and white color is showing down-regulated UGTs. Different colors are showing each phylogenetic group of putative UGTs.

A high expression of putative UGTs in *B. oleracea* was observed in root and pod tissues in comparison to the leaf and flower tissues (Fig. 9). A high expression of *Bo3g039900*, *Bo2g098930*, *Bo1g007610*, *Bo3g018000*, *Bo3g091760*, and *Bo9g090770* genes was observed in root tissues (Fig. 9). *Bo9g169250*, *Bo5g124870*, *Bo1g056770*, *Bo1g007610*, *Bo3g078830* were dominantly expressed in pod tissue of *B. oleracea* (Fig. 9). Two UGTs i.e. *Bo3g091760* and *Bo1g056770* were maximally expressed only in flower, while *Bo1g007610*, *Bo4g031150*, *Bo4g049480*, *Bo4g030990*, and *Bo9g090770* genes were expressed in leaf tissues (Fig. 9). The UGTs from the following phylogenetic groups (E, D, H, L, K, A and M) were abundantly expressed in calcium limited seedling tissues of root, leaf, and flower (Fig. 9). Some key regulated UGTs were following i.e. *Bo3g078820*, *Bo2g098930*, *Bo7g021600*, *Bo9g176810*, *Bo1g056770*, *Bo4g030990*, *Bo9g090770*, and *Bo5g008070* against low calcium treatment (Fig. 9).

**Figure 10:**
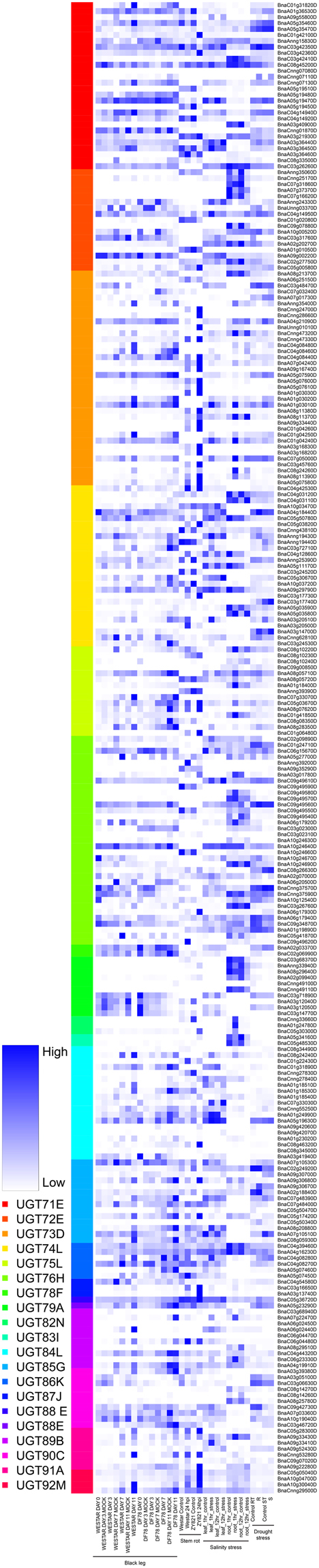
Differential expression of up and down regulated UGTs of *B. napus* in response to biotic stresses (blackleg and stem rot disease) and abiotic stresses (salinity and drought stress). Dark blue color is showing up-regulated UGTs and white color is showing down-regulated UGTs. Different colors are showing each phylogenetic group of putative UGTs in *B. napus*.

In *B. napus*, UGTs from phylogenetic groups (E, D, L, J, K, I and A) were having the most pathogen responsive genes against *Leptosphaeria maculans* infestation. In resistant genotype (DF78), a low expression of maximum UGTs was observed upon *L. maculans* infestation after 0, 3, 7 and 11 days treatment (Fig. 10). *BnaA05g19470D*, *BnaA09g00220D*, *BnaC04g08440D*, *BnaC04g12860D* and *BnaC03g71890D* UGTs increased their expression in Westar genotype (susceptible) upon infestation (Fig. 10). Only two UGT genes i.e. *BnaCnng01870D* and *BnaC01g31820D* were found to be upregulated in DF78 genotype upon infection (Fig. 10).

Again, a decrease in expression of maximum UGTs in Westar and ZY821 (resistant genotype) upon sclerotinia stem rot infestation after 24 hpi (Fig. 10). UGTs from phylogenetic groups (A, B, C, D, E, L, J, K, and M) showed a variation in their expression upon infestation. *BnaA03g36460D*, *BnaCnng24700D*, *BnaC05g03820D*, *BnaA09g30680D*, *BnaA05g07450D*, *BnaC06g04480D*, *BnaA03g39380D*, *BnaC05g28300D*, and *BnaA10g04700D* were the only UGTs increased their expression in both genotypes against stem rot in *B. napus* (Fig. 10).

The salt treatment in *B. napus* decreased the putative UGTs expression at different levels in leave and root tissues (Fig. 10). A 12 h salt treated leaves and root tissues abruptly decreased the expression of maximum number of UGTs. *BnaA03g36450D*, *BnaA07g04240D*, *BnaC03g72710D*, *BnaA08g28350D*, and *BnaC05g05040D* UGTs showed an increase in their expression upon 1 h salt treatment only, while the same genes were showing a decreased expression upon 12 h stress treatment (Fig. 10).

The drought treatment of 200 g *L*^−1^ PEG-6000 in B07Y19 (resistant) and 07Y29 (susceptible) genotypes also resulted a decrease in maximum UGTs expression in both genotypes (Fig. 10). *BnaC04g14940*, *BnaC04g14950D*, *BnaCnng47330D*, *BnaA04g18440D*, *BnaC09g49560D*, *BnaC04g08280D* and *BnaC05g36720D* were up-regulated in B07Y19 upon drought treatment (Fig. 10). A decreased expression of the same genes was observed in 07Y29 upon treatment (Fig. 10).

## Discussion

A greater number of identified putative UGTs (251) in *B. napus*, compared to its diploid progenitors *B. rapa* (140 UGTs) and *B. oleracea* (154 UGTs), showed the direct effect of polyploidization in increasing the number of UGTs in polyploid crops (Supplementary Table S1). This effect has also been observed in *Gossypium hirsutum* (196 UGTs), compared to *G. raimondii* (142 UGTs) and *G. arboretum* (146 UGTs)^14^. Thus, we propose that polyploid crops (wheat, cotton, tobacco, peanut, apple, etc.) have diversified their secondary metabolite biosynthesis, as indicated by the increased number of UGT genes. Total transcriptome sharing of the putative UGTs with 0.32%, 0.26%, and 0.24% in *B. rapa*, *B. oleracea*, and *B. napus* indicates that UGTs constitute one of the largest multigene families in *Brassica*. A similar transcriptome sharing ratio of the putative UGT genes was also observed in *Arabidopsis* (0.22%) and *Linum usitatissimum* (0.31%)^10,12^. By employing the same tools as those used in the present study, the UDP-1 family has been investigated in several other species, such as *Prunus persica* (169 UGTs), *Malus x domesticaa* (254 UGTs), *Vitis vinifera* (184 UGTs*), Linum usitatissimum* (138 UGTs), *Glycine max* (149 UGTs), *Gossypium hirsutum* (196 UGTs), and *Populus trichocarpa* (191 UGTs; unpublished data), which showed a wide distribution of this family among vascular plants^12–17,45,50^. The conserved percentage of GC contents from 44.36% to 45% from the diploid to polyploid level, in all three *Brassica* species, indicate their conserved mode of multiplication from progenitors to cultivars^14^ (Supplementary Table S1). The same conservation pattern of putative UGTs with respect to leucine-rich proteins was also observed in soybean^51^ (Supplementary Table S1).

The 14 identified phylogenetic groups (A-N) of all the putative UGTs in the three *Brassica* species were similar to *Arabidopsis*, which indicates that this family has not expanded in terms of new phylogenetic groups after separation from *Arabidopsis*^10,11^ (Figs 1 and 2). The presence of newly identified O and P groups in higher plants such as maize, but their absence in the three *Brassica* species and *Arabidopsis* indicates that they have been lost at some stage during the evolution of these plants^13^ (Figs 1 and 2). An expansion of D, E, H, and L groups in all three *Brassica* species compared to *Arabidopsis*, indicates that multiple functions are associated with these groups of UGTs and they have a broad substrate specificity^13^. Groups B, C, and O were not as expanded as others, which suggests that they have a conserved substrate specificity^13^ (Figs 1 and 2). Surprisingly, group H expanded only in the three *Brassica* species and *Arabidopsis*, whereas it contracted in all other eudicots, indicating that these UGTs are more required in *Brassica* species but have limited functions in other plants (Figs 1 and 2). An expansion, conservation, and reduction of UGT genes in each phylogenetic group of the eudicots reflects the physiological challenges that plants have to overcome for survival on land^13^. The absence of group F was observed in *B. oleracea*, indicating that these UGTs have been lost during evolution, and the same results were found for *G. hirsutum*, where group C was found missing^14^.

Structural investigation of the PSPG motif in each phylogenetic group revealed the role of specific amino acid residues that are highly conserved at positions 1 (W), 4 (Q), 10 (H), 19 (H), and 44 (Q) (Fig. 1B). The occurrence of these specific amino acids at these positions in the sequence provides certain evolutionary and functional information that could be helpful for enzyme discovery^13^.

The involvement of all 545 putative UGTs in *Brassica* to manipulate 14 major KEGG pathways and 24 biological process suggests that this family is highly important for plants to cope with environment stimuli and cellular homeostasis (Fig. 3A,B). The maximum involvement of putative UGTs in flavonoid glucuronidation and their biosynthesis indicates their mode of action through flavonoid glycosylation i.e quercetin 3-O-, 7-O glycosylation etc. (Supplementary Fig. S2 and Supplementary Table S2). The maximum involvement of UGT genes in flavonoid glucuronidation, in response to toxic substances and ABA stimulus, indicates that they are unique modulators molecules in plants that interact with the environment^52^. Several genes for ABA glycosylation have already been functionally characterized in *Arabidopsis*, *Phaseolus vulgaris*, and *Vigna angularis*^41,44,53,54^. Their involvement in plant growth and development has already been verified, for instance, in the in seed and fiber development in soybean and cotton^14,16^.

A varied chromosomal distribution, but the same pattern of chromosome sharing in *B. rapa* AA genome to *B. napus* AA genome, and *B. oleracea* CC genome to *B. napus* CC genome, reflects the occurrence of recent gene duplication events and close phylogenetic relationships, which is consistent with the findings for *Arabidopsis*, soybean, and cotton UGTs^10,14,16^. The expansion of the UGT gene family is primarily due to WGD events^12,16^. Tandemly duplicated UGT genes in *B. rapa* and *B. oleracea* suggest that chromosomal rearrangements took a part in the versatility of plant secondary metabolism, and the same results was observed in mustard family, where 45% and 48% of the glucosinolate UGT loci in *Arabidopsis* and *Aethionema* descend from tandem duplication^55^ (Supplementary Fig. S3). All the duplicated gene pairs had a Ka/Ks ratio <1, indicating that UGT genes might have preferentially conserved function and structure under selection pressure^11,56^ (Table 1). The sixteen pairs of diverged duplication pairs of UGT genes between *B. rapa* and *B. oleracea* around 1 to 9 MYA indicate a recent divergence during *Brassica* triplication events (5–9 MYA)^57^ (Table 1). The six identified duplicated pairs duplicated around 13.65 to 21.27 MYA, indicating that these duplications occurred during the divergence of Chinese cabbage and *Arabidopsis*, around 9.1 to 16.1 MYA^45,58^ (Table 1).

The maximum number of UGTs retained in LF subgenome in all three *Brassica* species suggests that the fractionation is biased^11^ (Supplementary Table S3). The minimum number of UGTs in MF (MF1 and Mf2) subgenomes reflected the round of gene loss in these subgenomes^11^. It seems that LF experienced one round of gene loss in UGTs and retained more genes than MF1 and MF2, which experienced two rounds of gene loss^47^. Some UGTs were not contained in all three subgenomes, suggesting that these UGTs evolved individually and later became a part of the genome. The maximum number of duplicated regions were observed in MF1 and MF2 UGTs in *Brassica*, suggesting that these genes remained highly conserved during subgenome fractionation and rounds of genome duplication (Fig. 5)^47^.

An increased number of intron-containing UGTs in *B. napus*, compared to *B. rapa* and *B. oleracea*, suggested that polyploidization increased the proportion of introns during the hybridization of genomes (Supplementary Table S4). The results are in line with those on cotton polyploidization, where duplicated genes were found to evolve independently after the polyploid formation^59^. Conserved intron between (150–200 amino acids) was the most widespread and oldest intron observed in most members of the different phylogenetic groups in all three *Brassica* species (Supplementary Fig. S4). The same intron insertion was observed in *Z. mays*, *P. persica*, flax, soybean, and cotton UGTs^10,14–17^. Most of the intron insertions were observed in phase 1, across all three compared *Brassica* gene structures, suggesting that the majority of conserved introns are ancient elements and that their phases remain stable^13,14,17^ (Supplementary Fig. S4). Intron size within each phylogenetic group appears to be variable (Table S4), suggesting that the intron size has been gene-specific during evolution^13^.

The maximum expression of UGTs in callus, flower root, and silique suggested their active involvement in phytohormones, which are highly active in developing tissues (Supplementary Fig. S4A). The same results were observed in cotton and soybean, where the maximum expression of these genes was observed in developing seeds and fiber^14,16^. The individual gene expression of each UGT in a particular tissue reflected their specific role in various plant parts. The maximum expression of UGTs at the senescence stage in *Arabidopsis* suggested their involvement in the biological ageing of plant tissues, and the results were supported by up-regulated UGT genes at the senescence stage in cotton^60^ (Supplementary Fig. S5B).

To date, UGT genes have been well studied with respect to their functional role in the hypersensitive response against *P. syringae*, *F. graminearum* mycotoxin inactivation, DON resistance from *Fusarium* head blight, in tomato, *Arabidopsis*, wheat, and rice^24,26,61–64^. The maximum number of downregulated UGTs genes in response to infection with *P. brassicae* at early and late infection stages suggested their possibly role in SA and JA crosstalk and glycosylation^24–26^ (Supplementary Fig. S6A,B). A very few number of up-regulated UGT genes upon infection to *P. brassicae* suggested that these genes are also involved in some metabolite glycosylation, which is indirectly involved in pathogen resistance.

A diverse range of expression was observed in 118 UGTs of *Arabidopsis*, upon different phytohormone treatments (Fig. 6). To date, several UGT genes have been functionally characterized as the glycoconjugates of phytohormones in *Arabidopsis*, for example *UGT74D1*, *UGT74B1*, UGT84B1 (IAA glycosylation), *UGT87A2*, *UGT75D1*, *UGT71C5*, *UGT71B7*, *UGT71B8*, *UGT71B6* (ABA glycosylation), *UGT75D1*, *UGT74E2*, (Indole-3-butyric acid (IBA) glycosylation), *UGT73C5*, *UGT73C6* (brassinosteroids glycosylation), *UGT85A1*, *UGT76C1*, *UGT76C2* (zeatin glycosylation), *UGT74F1*, *UGT74F2*, *UGT73B3*, *UGT73B5* (SA glycosylation), and *UGT76B1* (SA-JA crosstalk)^38,42–44,53,64–71^. Thus, we propose that, in group L, UGTs for IAA, SA, and IBA glycosylation, in group E, UGTs for ABA glycosylation, group D UGTs for brassinosteroids and SA glycosylation, group H for zeatin glycosylation and SA-JA crosstalk in all three *Brassica* species.

The differentially up-regulated *UGT73B3*, *UGT73B4*, and *UG73B5* genes in response to all the studied pathogens suggest that they are the key genes regulated under all pathogen infection in *Arabidopsis* (Fig. 7). They belong to phylogenetic group D, the members of which preferably glycosylate the SA (pathogen responsive hormone)^72^. In tomato, *UGT73B3* and *UGT73B5* have been functionally characterized against the *P. syringae* hypersensitive response, based on SA-dependent induction^24^. In wheat, *UGT73C5* has been successfully transformed from *Arabidopsis* to inactivate the deoxynivalenol mycotoxin against *Fusarium* head blight^61^. Thus, we propose that the D group UGTs are key regulatory genes against pathogen response in all three *Brassica* species. The maximum up-regulation of *UGT71C3*, *UGT74E2*, and *UGT85A1* in response to *A. brassicicola* and *B. graminis* also confirmed their involvement in IBA and zeatin glycosylation^37,38^. Highly responsive *UGT87A1* against *P. cucumerina* infection in *Arabidopsis* suggested the ABA glycosylation of this gene against the pathogen^53^.

In *Arabidopsis*, *UGT85U1* and *UGT85V1* for salt and oxidative stress, *UGT73B5* for oxidative stress, *UGT84A1*, *A2*, *A3*, and *A4* for UV-B radiation stress, *UGT85A5* for salt tolerance, *UGT73B2* for oxidative stress, and *UGT74E2* for water stress, have already been functionally characterized^26,36–38,61,72^ (Fig. 8). Thus, we propose that a role is played by *UGT73C1*, *UGT78D3*, *UGT79B2*, *UGT84A3*, and *UGT90A1* in cold resistance, *UGT73B3*, *UGT73B5*, *UGT73C1*, *UGT74E2*, and *UGT79B1* in drought tolerance, *UGT79B2*, *AT4G31780*, *UGT85A1*, *UGT85A2*, *UGT86A2*, and *UGT90A1* in heat tolerance, *UGT71B1*, *UGT71B2*, *UGT71B7*, *UGT74D1*, *UGT76E1*, *UGT76F2*, *UGT78D1*, *UGT87A1*, *UGT88A1*, *UGT73B3*, *UGT73B5*, and *UGT73B6* in oxidative stress response, *UGT71B1*, *UGT71C1*, *UGT71C2*, *UGT71B1*, *UGT71D1*, *UGT72B2*, *UGT74D1*, *UGT74F2*, *UGT76E9*, *UGT84A2*, and *UGT84A4* in salt tolerance, and *UGT75C1* and *UGT78D1* in chilling resistance, in *Arabidopsis* and all three *Brassica* species.

Until now, few reports have been published about functional characterization of UGT genes against stress responsiveness in all three-brassica species. The availability of Arabidopsis data provides sufficient evidence about UGTs importance in plant physiology and a maximum homology of all three *Brassica* UGTs with *Arabidopsis* is another opportunity to validate this data in brassica species (Supplementary Fig. S1). Recently, a *BrUGT74B1* was found to be involved in phytoalexins biosynthesis which have an important role in plant disease resistance^73^. Previously, an overexpression of the same genes in *B*. *napus* increased the aliphatic and indolic glucosinolates levels by 1.7 and 1.5 folds in leaves against *Sclerotinia sclerotiorum* and *Botrytis cinereal* pathogens^74^. In general, a decreased expression by maximum UGTs under biotic and abiotic stresses in all the observed datasets suggested that glycosylation mechanism is hindered by putative UGTs upon stress in *B. napus* (Fig. 10). Based on UGTs expression against various biotic and abiotic stresses, some key UGTs and their close homologs could be the potential candidates for functional characterization i.e. *BnaCnng01870D* and *BnaC01g31820D* (blackleg resistance*), BnaC01g42100D BnaC03g42360D, BnaA08g11370D, BnaA08g28350D* (stem rot resistance), *BnaA02g20270D*, *BnaC07g05000D*, and *BnaA08g20880D* (salt stress resistance), *BnaC04g14940*, *BnaC04g14950D*, *BnaCnng47330D*, *BnaA04g18440D*, *BnaC09g49560D*, *BnaC04g08280D* and *BnaC05g36720D* (drought stress resistance) (Fig. 10).

## Methods

### Data resources used

One hundred and eighteen UGT sequences of *Arabidopsis* were retrieved from (http://www.p450.kvl.dk/UGT.shtml). Genomic, proteomic, and cDNA sequences, as well as the annotation data for *B. rapa*, *B. oleracea*, and *B. napus* was downloaded from BioMart Ensemble plants (https://plants.ensembl.org/index.html) and Uniprot (http://www.uniprot.org/). The Hidden Markov Model (HMMER) (http://www.ebi.ac.uk/Tools/hmmer/) web server was used to retrieve the genes containing UDPGT (PF00201.17) domain with significant hits in all three *Brassica* species. The BRAD database (http://brassicadb.org/brad/) and the Bolbase database (http://ocri-genomics.org/bolbase) were used for gene syntenic analysis. Expression Array and RNA Sequencing data were obtained from the Gene Expression database with the following accession numbers: GSE43245 in *B. rapa* for expression in tissues, GSE74044 for the clubroot disease, GSE51363 for expression against Methyl JA in *B. napus*. Some additional datasets were personally requested and obtained for the club root disease RNA-seq from the following authors^48^.

### Identification of Putative UGTs

In the reference proteomes of all three *Brassica* species, UDPGT domain encoding genes were identified through a PHMMER profile corresponding to the PFAM 00201.17 domain, using HMMER web search restricted by taxonomy and significant cut off E- values (0.01 for sequence and 0.03 for hit). The selected protein sequences were screened through 44 amino acid-based conserved PSPG motifs, high quality sequences were aligned using the MUSCLE alignment tool in MEGA7 (http://www.megasoftware.net/). These quality sequences were used to construct the specific UGT profiles for all three *Brassica* species, using the hmmbuild module in the HMMER program. With these models, the final datasets of the UGT genes were identified from each *Brassica* proteome. The gene name, chromosomal location, % GC content, peptide length, and mass data were obtained from the Biomart Ensemble Plants database. Gene ontology (metabolic process, integral components, and transferase activity) and enzyme class data were retrieved using the ID mapping tool in Uniprot database. Amino acid composition of all the putative UGTs was retrieved using the data explorer tool in MEGA7 software.

### Phylogenetic Analysis and Comparison

The downloaded sequences were aligned with MUSCLE using the MEGA7 software. Sequences that were too short, too divergent, or too long were removed from the input file after the initial alignment, and the remaining sequences were then re-aligned. The obtained alignment file contained only those sequences similar to the desired PSPG motif. Phylogenetic analysis was performed using the Neighbor-joining statistical method with 1000 bootstrap replicates in MEGA7. A 100% data coverage was used to construct the phylogeny. The phylogenetic tree was visualized using the iTOL website (http://itol.embl.de/). To compare the phylogenetic groups of the putative UGTs in all three *Brassica* species, we collected the published data on the number of putative UGTs and the number of phylogenetic groups in *Arabidopsis*^10^.

### KEGG Pathway Mapping and Go Annotation

Gene ontology and KEGG pathway mapping of all the putative UGTs in the three *Brassica* species were conducted using the Blast2Go tool (https://www.blast2go.com/). Individual UGTs from each phylogenetic group in all three *Brassica* species were blasted against *Arabidopsis* in the NCBI database following with mapping and annotation. This analysis provided us the sequence similarity with *Arabidopsis* and the Go annotation (molecular function, and biological process) for each group and gene. KEGG pathway mapping based on phylogenetic groups provided us with enzyme-specific pathway information, EC code, and the responsible genes in each group and each UGT gene of all three *Brassica* species.

### Chromosomal distribution, Duplication, and Divergence

The physical location of each UGT on the chromosomes was retrieved using the start and stop positions of the genes taken from BioMart. Mapchart 2.2 was used to visualize the UGT gene distribution on each chromosome in all three *Brassica* species. A gene cluster was defined as two or more copies located in a chromosomal region of length <200 kb^75^. A lot is known about the dominant pattern of UGT duplication by segmental duplication and WGD in maize and peach^15,16^. To detect the tandem duplication pattern of the putative UGTs, we used the Plant Tandem Duplicated Genes database (http://ocri-genomics.org/PTGBase/). Tandem repeats of each gene were retrieved using the Gene ID as the query in that specific species cluster. The cluster name and its graphical representation was recorded and saved.

To estimate the divergence of the duplicated UGT genes between *B. rapa* (AA) and *B. oleracea* (CC) genomes, the duplicated pairs were detected in the Plant gene duplication database (http://chibba.agtec.uga.edu/duplication/) using the locus IDs as a search tool and the Ka (non-synonymous)/Ks (synonymous rate) values were recorded for each duplicated pair in a 100-kb display range. The R-value was taken as 1.5 × 10^−8^ synonymous substitutions per site per year in the case of dicotyledonous plants for MYA calculation^76^.

### Gene Synteny and subgenomic Fractionation of the putative UGTs

Syntenic gene analysis for the three *Brassica* species and *Arabidopsis* was conducted using the webtool, syntenic gene, in the Brad database (http://brassicadb.org/brad/). All the four species were selected and the gene IDs of *B. rapa* were used to retrieve information such as tPCK (Chromosome of translocation Proto-Calepineae Karyotype, ancestral genome of *Brassica* species), block (A, B, C, etc), LF, MF1, MF2, and gene IDs^58^. Graphical representation was achieved using the Circoletto webtool (http://tools.bat.infspire.org/circoletto/) by providing the FASTA sequences in each block for all three *Brassica* species and *Arabidopsis*.

### Exon/Intron organization of the putative UGTs in *Brassica*

The exon/intron organization for each phylogenetic group was illustrated using the Gene structure display server (GSDS) program (http://gsds.cbi.pku.edu.cn/), by aligning the coding and genomic sequences obtained from BioMart. Introns were classified based on structure, phase, length, and number. A UGT intron map was constructed in accordance with a previously established method^12,16,17^. The splice sites of the intron-containing UGTs were mapped on all aligned sequences of intron-containing UGT peptides using the PIECE web tool (https://wheat.pw.usda.gov/piece/). Intron distribution graph was built for each phylogenetic group on the basis of the introns contained in each group.

### Transcriptome analysis for tissue-specific expression

To check tissue-specific expression of the putative UGTs in all three *Brassica* species, the RNA-Seq data (GSE43245) of *B. rapa* was selected and the expression of the putative UGTs in different tissues, such as callus, flower, leaves, roots, silique, and stem, was obtained and analyzed according to the phylogenetic groups. A heatmap of the data was generated using the distance function (Euclidean) and hierarchical clustering (Average) on the Heatmapper website (http://heatmapper.ca/). To compare tissue-specific expression data of *B. rapa*, we analyzed the RNA-Seq and microarray data for expression of 118 UGTs across 10 developmental stages and 105 anatomical parts, using the Genevestigator database.

### Transcriptome analysis against *Plasmodiophora brassicae*

To infer the role of the putative UGTs against the obligate biotrophic protist, *P. brassicae*, in *B. rapa* and *Arabidopsis*, RNA seq data were obtained at two timepoints: an early timepoint, at which the pathogen had colonized the root, and a later timepoint, at which more than 60% of the host root cells were colonized and the root morphology was drastically altered^48^. In *Arabidopsis*, RNA-Seq data at early stages of infection and within a relatively short period of 24 and 48 h post-inoculation (hpi) were retrieved from a previous study^48^. The retrieved RNA-seq data against *P. brassicae* in *Arabidopsis* was compared with the obtained RNA-seq data from near-isogenic lines carrying clubroot-resistant and susceptible alleles in *B. rapa* in response to *P. brassicae* during the early infection stages^49^. Heatmaps were generated to understand the graphical representation of gene expression.

### Differential expression of Phytohormone Glycosylation

The glycosylation of phytohormones is an essential mechanism to control the level of active hormones in a cell. To check the expression of individual UGTs against auxins, cytokinins, SA, and JA, microarray datasets were obtained from GENEVESTIGATOR^®^ (https://genevestigator.com/gv/) for *Arabidopsis*, and the results were further confirmed using *B. rapa* RNA-seq data (Only MeJA data available). Genes were declared to be differentially expressed if they show a fold-change of at least 1.5 and also satisfied *p* < 0.05 after adjustment for multiple testing^77,78^. Thus, we generated the heatmaps with fold-change value of 1.5 and *p* < 0.05 against all the treatments. Down- and up-regulated expressed genes were shown to propose the functional characterization of UGTs for phytohormone glycosylation.

### Differential expression against Biotic and Abiotic stresses in *Arabidopsis*

To contribute to the existing knowledge for future studies and examine the link of glycosylation phenomena with various biological processes in the plants, we checked the differential expression of these genes against various biotic and abiotic stress treatments. By using the fold-change value of 1.5 and *p* < 0.05, we identified the differentially expressed genes against *A. brassiciola, B. graminis, E. coli, G. cichoracearum, G. orontii, H arabidopsidis, P. cuccumerina, P. syringae, R. solani*, *and X campestris* pathogens, as well as cold, drought, heat, hypoxia, osmotic, salt, and wounding stresses. Heatmaps of the down- and up-regulated UGTs were generated using Genevestigator.

### Differential expression against Biotic and Abiotic stresses in *B. oleracea* and *B. napus*

The following SRA datasets PRJNA227258^79^ (calcium- limited seedlings expression in 102043 and 107140 genotypes) and SRP040796^80^ (leaf, flower, root and pod expression) were obtained and analyzed by Galaxy (https://usegalaxy.org/) RNA-seq analysis webtool in *B. oleracea* for differential gene expression in tissues and calcium limited plants. The UGTs involvement against abiotic stresses (drought and salt) and biotic stresses (blackleg and stem rot) in polyploid *B. napus* was evaluated by analyzing the following SRA datasets i.e. SRP051461^81^ (drought), SRP028575^82^ (salinity), GSE77723^83^ (blackleg) and GSE81545^84^ (stem rot). Hence, we generated the individual heatmaps in *B. oleracea* and *B. napus* using the fold-change value of 1.5 and *p* < 0.05.

### Data Availability statement

All data generated or analyzed during this study are included in this published article (and its supplementary information files).

## Electronic supplementary material

Supplementary Figures

Supplementary Table S1

Supplementary Table S2

Supplementary Table S3

Supplementary Table S4

Supplementary Table S5

Supplementary Table S6

## References

[1] Sharma A, Li X, Lim YP (2014). Comparative genomics of Brassicaceae crops. Breed Sci.

[2] Schmidt, R. & bancroft, I. Genetics and genomics of the Brassicaceae. Plant genetics and genomics: crops and models. *Springer* **9** (2011).

[3] Bowles D, Lim EK, Poppenberger B, Vaistij FE (2006). Glycosyltransferases of lipophilic small molecules. Annu Rev Plant Biol.

[4] Jones P, Vogt T (2001). Glycosyltransferases in secondary plant metabolism: tranquilizers and stimulant controllers. Planta.

[5] Vogt T, Jones P (2000). Glycosyltransferases in plant natural product synthesis: characterization of a supergene family. Trends Plant Sci.

[6] Coutinho PM, Deleury E, Davies GJ, Henrissat B (2003). An evolving hierarchical family classification for glycosyltransferases. J Mol Biol.

[7] Hughes J, Hughes MA (1994). Multiple secondary plant product UDP-glucose glucosyltransferase genes expressed in cassava (Manihot esculenta Crantz) cotyledons. DNA Seq..

[8] Lorenc-Kukula K (2004). Glucosyltransferase: the gene arrangement and enzyme function. Cell Mol Biol Lett.

[9] Offen W (2006). Structure of a flavonoid glucosyltransferase reveals the basis for plant natural product modification. Embo j.

[10] Li Y, Baldauf S, Lim EK, Bowles DJ (2001). Phylogenetic analysis of the UDP-glycosyltransferase multigene family of Arabidopsis thaliana. J Biol Chem.

[11] Yu J, Hu F, Dossa K, Wang Z, Ke T (2017). Genome-wide analysis of UDP-glycosyltransferase super family in Brassica rapa and Brassica oleracea reveals its evolutionary history and functional characterization. BMC Genomics.

[12] Barvkar VT, Pardeshi VC, Kale SM, Kadoo NY, Gupta VS (2012). Phylogenomic analysis of UDP glycosyltransferase 1 multigene family in Linum usitatissimum identified genes with varied expression patterns. BMC Genomics.

[13] Caputi L, Malnoy M, Goremykin V, Nikiforova S, Martens S (2012). A genome-wide phylogenetic reconstruction of family 1 UDP-glycosyltransferases revealed the expansion of the family during the adaptation of plants to life on land. Plant J.

[14] Huang J (2015). Genome-wide analysis of the family 1 glycosyltransferases in cotton. Mol Genet Genomics.

[15] Li Y (2014). Genome-wide identification and phylogenetic analysis of Family-1 UDP glycosyltransferases in maize (Zea mays). Planta.

[16] Mamoon Rehman H (2016). Genome-wide analysis of Family-1 UDP-glycosyltransferases in soybean confirms their abundance and varied expression during seed development. J. Plant Physiol..

[17] Wu B (2017). Genome-Wide Identification, Expression Patterns, and Functional Analysis of UDP Glycosyltransferase Family in Peach (Prunus persica L. Batsch). Front Plant Sci.

[18] Campbell JA, Davies GJ, Bulone V, Henrissat B (1997). A classification of nucleotide-diphospho-sugar glycosyltransferases based on amino acid sequence similarities. Biochem J.

[19] Ross J, Li Y, Lim E, Bowles DJ (2001). Higher plant glycosyltransferases. Genome Biol.

[20] Yonekura-Sakakibara K, Hanada K (2011). An evolutionary view of functional diversity in family 1 glycosyltransferases. Plant J.

[21] Cantarel BL (2009). The Carbohydrate-Active EnZymes database (CAZy): an expert resource for Glycogenomics. Nucleic Acids Res.

[22] Park BH, Karpinets TV, Syed MH, Leuze MR, Uberbacher EC (2010). CAZymes Analysis Toolkit (CAT): web service for searching and analyzing carbohydrate-active enzymes in a newly sequenced organism using CAZy database. Glycobiology.

[23] Le Roy J, Huss B, Creach A, Hawkins S, Neutelings G (2016). Glycosylation Is a Major Regulator of Phenylpropanoid Availability and Biological Activity in Plants. Front Plant Sci.

[24] Langlois-Meurinne, M., Gachon, C. M. M. & Saindrenan, P. Pathogen-responsive expression of glycosyltransferase genes UGT73B3 and UGT73B5 is necessary for resistance to Pseudomonas syringae pv tomato in Arabidopsis. *Plant Physiol*., 10.1104/pp.105.067223 (2005). 10.1104/pp.105.067223 PMC1310567 16306146

[25] von Saint Paul V (2011). The Arabidopsis glucosyltransferase UGT76B1 conjugates isoleucic acid and modulates plant defense and senescence. Plant Cell.

[26] Song JT (2008). Overexpression of AtSGT1, an Arabidopsis salicylic acid glucosyltransferase, leads to increased susceptibility to Pseudomonas syringae. Phytochemistry.

[27] Boachon B (2014). Role of two UDP-Glycosyltransferases from the L group of arabidopsis in resistance against pseudomonas syringae. Eur. J. Plant Pathol..

[28] George Thompson AM, Iancu CV, Neet KE, Dean JV, Choe J (2017). Differences in salicylic acid glucose conjugations by UGT74F1 and UGT74F2 from Arabidopsis thaliana. Nat. Sci. Reports.

[29] Manoharan RK, Shanmugam A, Hwang I, Park J-I, Nou I-S (2016). Expression of salicylic acid-related genes in Brassica oleracea var. capitata during Plasmodiophora brassicae infection. Genome.

[30] Chong J (2002). Downregulation of a pathogen-responsive tobacco UDP-Glc:phenylpropanoid glucosyltransferase reduces scopoletin glucoside accumulation, enhances oxidative stress, and weakens virus resistance. Plant Cell.

[31] Matros A, Mock HP (2004). Ectopic expression of a UDP-glucose:phenylpropanoid glucosyltransferase leads to increased resistance of transgenic tobacco plants against infection with Potato Virus Y. Plant Cell Physiol.

[32] Konig S (2014). Soluble phenylpropanoids are involved in the defense response of Arabidopsis against Verticillium longisporum. New Phytol.

[33] Ha X, Koopmann B, von Tiedemann A (2016). Wheat Blast and Fusarium Head Blight Display Contrasting Interaction Patterns on Ears of Wheat Genotypes Differing in Resistance. Phytopathology.

[34] Poppenberger B (2003). Detoxification of the Fusarium mycotoxin deoxynivalenol by a UDP-glucosyltransferase from Arabidopsis thaliana. J Biol Chem.

[35] Hettwer K (2016). Dynamic metabolic changes in seeds and seedlings of Brassica napus (oilseed rape) suppressing UGT84A9 reveal plasticity and molecular regulation of the phenylpropanoid pathway. Phytochemistry.

[36] Ahrazem O (2015). Ectopic expression of a stress-inducible glycosyltransferase from saffron enhances salt and oxidative stress tolerance in Arabidopsis while alters anchor root formation. Plant Sci.

[37] Sun YG (2013). Ectopic expression of Arabidopsis glycosyltransferase UGT85A5 enhances salt stress tolerance in tobacco. PLoS One.

[38] Tognetti VB (2010). Perturbation of indole-3-butyric acid homeostasis by the UDP-glucosyltransferase UGT74E2 modulates Arabidopsis architecture and water stress tolerance. Plant Cell.

[39] Vanderauwera S (2005). Genome-wide analysis of hydrogen peroxide-regulated gene expression in Arabidopsis reveals a high light-induced transcriptional cluster involved in anthocyanin biosynthesis. Plant Physiol.

[40] Hajihashemi S, Geuns JM (2016). Gene transcription and steviol glycoside accumulation in Stevia rebaudiana under polyethylene glycol-induced drought stress in greenhouse cultivation. FEBS Open Bio.

[41] Gilbert MK (2013). A transcript profiling approach reveals an abscisic acid-specific glycosyltransferase (UGT73C14) induced in developing fiber of Ligon lintless-2 mutant of cotton (Gossypium hirsutum L.). PLoS One.

[42] Liu Z (2015). UDP-glucosyltransferase71c5, a major glucosyltransferase, mediates abscisic acid homeostasis in Arabidopsis. Plant Physiol.

[43] Priest DM, Jackson RG, Ashford DA, Abrams SR, Bowles DJ (2005). The use of abscisic acid analogues to analyse the substrate selectivity of UGT71B6, a UDP-glycosyltransferase of Arabidopsis thaliana. FEBS Lett.

[44] Zhang GZ (2016). Ectopic expression of UGT75D1, a glycosyltransferase preferring indole-3-butyric acid, modulates cotyledon development and stress tolerance in seed germination of Arabidopsis thaliana. Plant Mol Biol.

[45] Song C (2015). Functional Characterization and Substrate Promiscuity of UGT71 Glycosyltransferases from Strawberry (Fragaria x ananassa). Plant Cell Physiol.

[46] Kwon Kyoo K (2012). Isolation and functional characterization of BrUGT gene encoding a UDP-glycosyltransferase from Chinese cabbage (Brassica rapa). J. Plant Biotechnol..

[47] Cheng F, Wu J, Wang X (2014). Genome triplication drove the diversification of Brassica plants. Hortic. Res..

[48] Zhao Y (2017). Transcriptome Analysis of Arabidopsis thaliana in Response to Plasmodiophora brassicae during EarlyInfection. Front. Microbiol..

[49] Chen T (2016). Arabidopsis Mutant bik1 Exhibits Strong Resistance to Plasmodiophora brassicae. Front Physiol.

[50] Bonisch F (2014). Activity-based profiling of a physiologic aglycone library reveals sugar acceptor promiscuity of family 1 UDP-glucosyltransferases from grape. Plant Physiol.

[51] Rehman HM (2016). Genome-wide analysis of Family-1 UDP-glycosyltransferases in soybean confirms their abundance and varied expression during seed development. J. Plant Physiol..

[52] Mierziak J, Kostyn K, Kulma A (2014). Flavonoids as important molecules of plant interactions with the environment. Molecules.

[53] Li P (2017). The Arabidopsis UGT87A2, a stress-inducible family 1 glycosyltransferase, is involved in the plant adaptation to abiotic stresses. Physiol Plant.

[54] Palaniyandi SA, Chung G, Kim SH, Yang SH (2015). Molecular cloning and characterization of the ABA-specific glucosyltransferase gene from bean (Phaseolus vulgaris L.). J. Plant Physiol..

[55] Hofberger JA, Lyons E, Edger PP, Chris Pires J, Eric Schranz M (2013). Whole genome and tandem duplicate retention facilitated glucosinolate pathway diversification in the mustard family. Genome Biol Evol.

[56] Nekrutenko A, Makova KD, Li WH (2002). The K(A)/K(S) ratio test for assessing the protein-coding potential of genomic regions: an empirical and simulation study. Genome Res.

[57] Woodhouse MR (2014). Origin, inheritance, and gene regulatory consequences of genome dominance in polyploids. Proc Natl Acad Sci USA.

[58] Wang X (2011). The genome of the mesopolyploid crop species Brassica rapa. Nat Genet.

[59] Cronn RC, Small RL, Wendel JF (1999). Duplicated genes evolve independently after polyploid formation in cotton. Proc. Natl. Acad. Sci..

[60] Lin M (2015). Global analysis of the Gossypium hirsutum L. Transcriptome during leaf senescence by RNA-Seq. BMC Plant Biol..

[61] Schweiger, W. *et al*. Functional characterization of two clusters of Brachypodium distachyon UDP-glycosyltransferases encoding putative deoxynivalenol detoxification genes. *Mol. Plant. Microbe. Interact*., 10.1094/MPMI-08-12-0205-R (2013). 10.1094/MPMI-08-12-0205-R 23550529

[62] Li X (2015). Transgenic Wheat Expressing a Barley UDP-Glucosyltransferase Detoxifies Deoxynivalenol and Provides High Levels of Resistance to Fusarium graminearum. Mol Plant Microbe Interact.

[63] Michlmayr H (2015). Biochemical Characterization of a Recombinant UDP-glucosyltransferase from Rice and Enzymatic Production of Deoxynivalenol-3-O-β-D-glucoside. Toxins (Basel)..

[64] Poppenberger B (2005). The UGT73C5 of Arabidopsis thaliana glucosylates brassinosteroids. Proc. Natl. Acad. Sci. USA.

[65] Tanaka K (2014). UGT74D1 catalyzes the glucosylation of 2-oxindole-3-acetic acid in the auxin metabolic pathway in Arabidopsis. Plant Cell Physiol.

[66] Grubb CD (2014). Comparative analysis of Arabidopsis UGT74 glucosyltransferases reveals a special role of UGT74C1 in glucosinolate biosynthesis. Plant J..

[67] Dong T (2014). Abscisic acid uridine diphosphate glucosyltransferases play a crucial role in abscisic acid homeostasis in Arabidopsis. Plant Physiol.

[68] Jackson RG (2001). Identification and biochemical characterization of an Arabidopsis indole-3-acetic acid glucosyltransferase. J Biol Chem.

[69] Sun, Y. G. *et al*. Ectopic Expression of Arabidopsis Glycosyltransferase UGT85A5 Enhances Salt Stress Tolerance in Tobacco. *PLoS One*, 10.1371/journal.pone.0059924 (2013). 10.1371/journal.pone.0059924 PMC3606239 23533660

[70] Husar S (2011). Overexpression of the UGT73C6 alters brassinosteroid glucoside formation in Arabidopsis thaliana. BMC Plant Biol.

[71] Hou B, Lim EK, Higgins GS, Bowles DJ (2004). N-glucosylation of cytokinins by glycosyltransferases of Arabidopsis thaliana. J Biol Chem.

[72] Mazel A, Levine A (2002). Induction of glucosyltransferase transcription and activity during superoxide-dependent cell death in Arabidopsis plants. Plant Physiol. Biochem..

[73] Klein AP, Sattely ES (2017). Biosynthesis of cabbage phytoalexins from indole glucosinolate. Proceedings of the National Academy of Sciences of the United States of America.

[74] Zhang Y (2015). Overexpression of Three Glucosinolate Biosynthesis Genes in Brassica napus Identifies Enhanced Resistance to Sclerotinia sclerotiorum and Botrytis cinerea. PLOS ONE.

[75] Voorrips RE (2002). MapChart: software for the graphical presentation of linkage maps and QTLs. J Hered.

[76] Koch MA, Haubold B, Mitchell-Olds T (2000). Comparative evolutionary analysis of chalcone synthase and alcohol dehydrogenase loci in Arabidopsis, Arabis, and related genera (Brassicaceae). Mol Biol Evol.

[77] Peart MJ (2005). Identification and functional significance of genes regulated by structurally different histone deacetylase inhibitors. Proc Natl Acad Sci USA.

[78] Raouf A (2008). Transcriptome analysis of the normal human mammary cell commitment and differentiation process. Cell Stem Cell.

[79] Kim, H. A. *et al*. High-Throughput Sequencing and De Novo Assembly of Brassica oleracea var. Capitata L. for Transcriptome Analysis. *PLOS ONE* **9**, 1–10 (2014). 10.1371/journal.pone.0092087 PMC3969326 24682075

[80] Parkin IAP (2014). Transcriptome and methylome profiling reveals relics of genome dominance in the mesopolyploid Brassica oleracea. Genome Biology.

[81] Wang, P. *et al*. Transcriptomic basis for drought-resistance in Brassica napus L. *Sci Rep* **7**, 10.1038/srep40532 (2017). 10.1038/srep40532 PMC5238399 28091614

[82] Yong H-Y (2014). Comparative Transcriptome Analysis of Leaves and Roots in Response to Sudden Increase in Salinity in Brassica napus by RNA-seq. BioMed research international.

[83] Becker MG (2017). Transcriptome analysis of the *Brassica napus*-Leptosphaeria maculans pathosystem identifies receptor, signaling and structural genes underlying plant resistance. The Plant journal: for cell and molecular biology.

[84] Girard IJ (2017). RNA sequencing of *Brassica napus* reveals cellular redox control of Sclerotinia infection. J Exp Bot.

