# Author Correction: Comparative genomic and transcriptomic analyses of Family-1 UDP glycosyltransferase in three *Brassica* species and *Arabidopsis* indicates stress-responsive regulation

**DOI:** 10.1038/s41598-018-24107-6

**Published:** 2018-04-13

**Authors:** Hafiz Mamoon Rehman, Muhammad Amjad Nawaz, Zahid Hussain Shah, Jutta Ludwig-Müller, Gyuhwa Chung, Muhammad Qadir Ahmad, Seung Hwan Yang, Soo In Lee

**Affiliations:** 1 Department of Biotechnology, Chonnam National University, Yeosu, Chonnam 59626 Korea; 2 Department of Arid Land Agriculture, King Abdul-Aziz University, Jeddah, Saudi Arabia; 3 Institut für Botanik, Technische Universität Dresden, 01062 Dresden, Germany; 4 Department of Plant Breeding and Genetics, Bahauddin Zakariya University, Multan, 6000 Pakistan; 5 Department of Agricultural Biotechnology, National Institute of Agricultural Sciences, Jeonju, 54874 Republic of Korea

Correction to: *Scientific Reports* 10.1038/s41598-018-19535-3, published online 30 January 2018

In this Article, Figure 7 is a duplication of Figure 6. The correct Figure 7 appears below as Figure 1.

**Figure 1:**
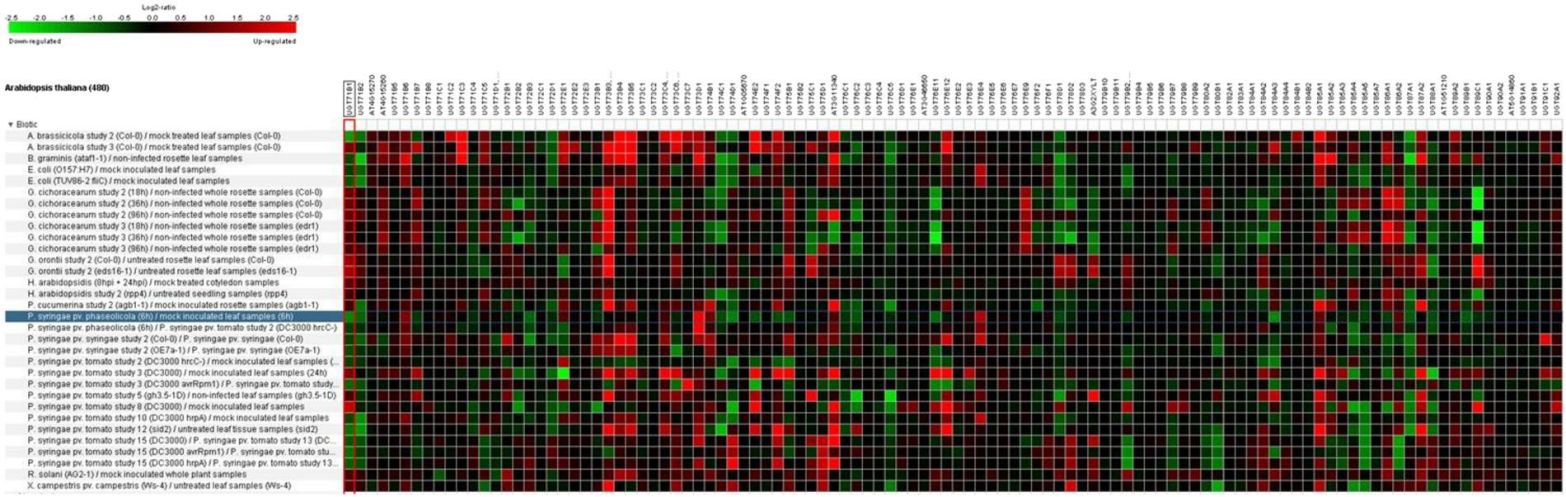
Differential expression of up and downregulated UGTs of *Arabidopsis* in response to different pathogen infestations (*A*. *brassiciola*, *B*. *graminis*, *E*. *coli*, *G*. *cichoracearum*, *G*. *orontii*, *H*. *arabidopsidis*, *P*. *cuccumerina*, *P*. *syringae*, *R*. *solani*, and *X*. *campestris*). Green color is showing down regulated UGTs and red color is showing up regulated UGTs.

