# Supplementary material for: Comparative genomic and transcriptomic analyses of Family-1 UDP glycosyltransferase in three Brassica species and Arabidopsis indicates stress-responsive regulation
Source: Sci Rep. 2018 Jan 30;8:1875. doi: 10.1038/s41598-018-19535-3 (PMC5789830; doi:10.1038/s41598-018-19535-3)
Supplement: Supplementary file 1 — Supplementary Figures [file 41598_2018_19535_MOESM1_ESM.doc]

**Comparative genomic and transcriptomic analyses of Family-1 UDP glycosyltransferase in three *Brassica* species and *Arabidopsis* indicates stress-responsive regulation**

**Hafiz Mamoon Rehman^1^, Muhammad Amjad Nawaz^1^, Zahid Hussain Shah^2^, Jutta Ludwig-Müller^3^, Gyuhwa Chung^1^, Muhammad Qadir Ahmad^4^, Seung Hwan Yang^1^*, Soo In Lee^5^***

^1^Department of Biotechnology, Chonnam National University, Yeosu, Chonnam 59626, Korea

^2^Department of Arid Land Agriculture, King Abdul-Aziz University Jeddah, Saudi Arabia

^3^Institut für Botanik, Technische Universität Dresden, 01062 Dresden, Germany

^4^Department of Plant Breeding and Genetics, Bahauddin Zakariya University, Multan-6000, Pakistan

^5^Department of Agricultural Biotechnology, National Institute of Agricultural Sciences Jeonju 54874, Republic of Korea

***Corresponding author (s)**

E-mail: ymichigan@jnu.ac.kr (SH. Yang)

E-mail: [silee@korea.kr](mailto:silee@korea.kr) (SI. Lee)


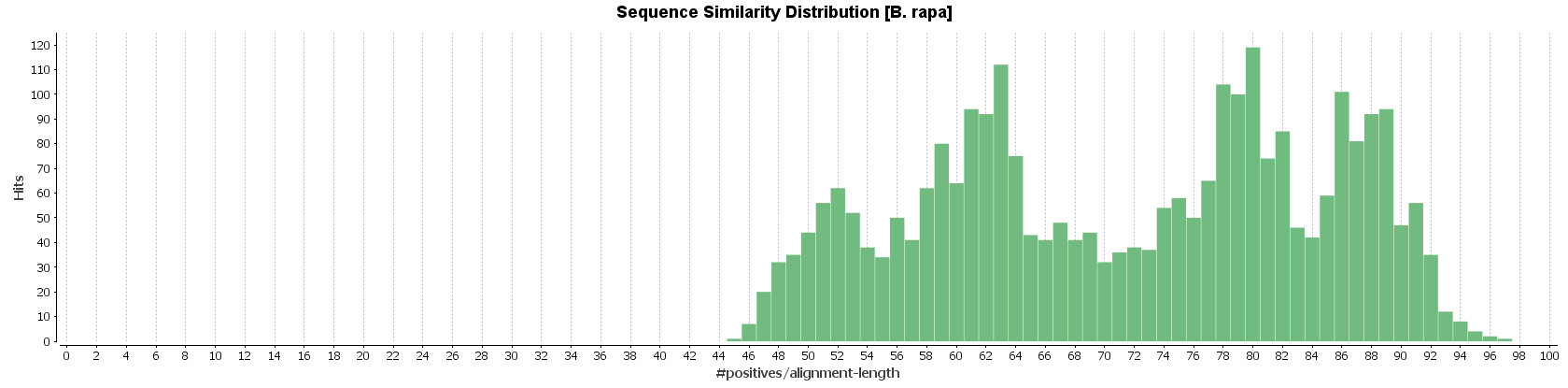

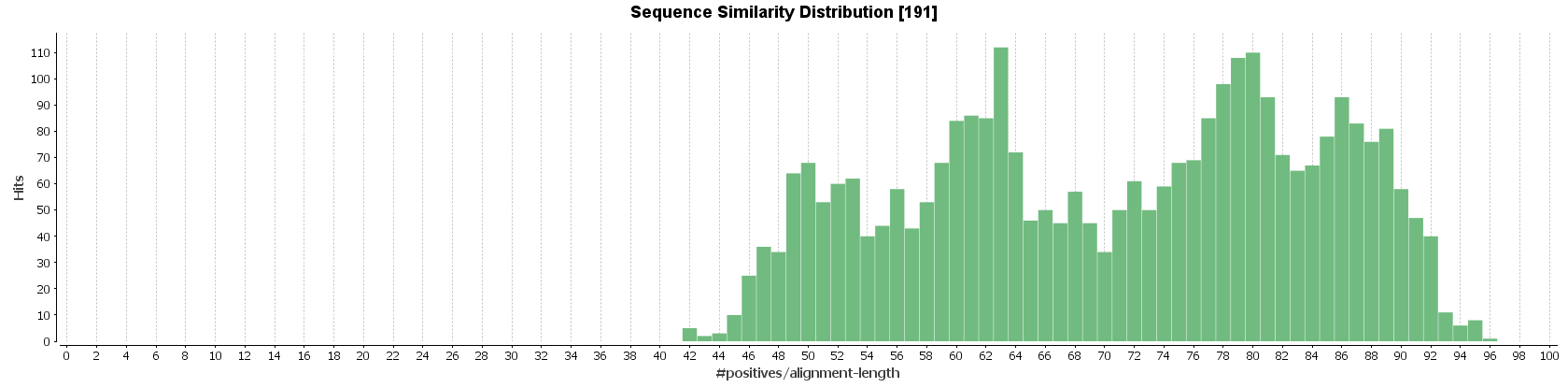

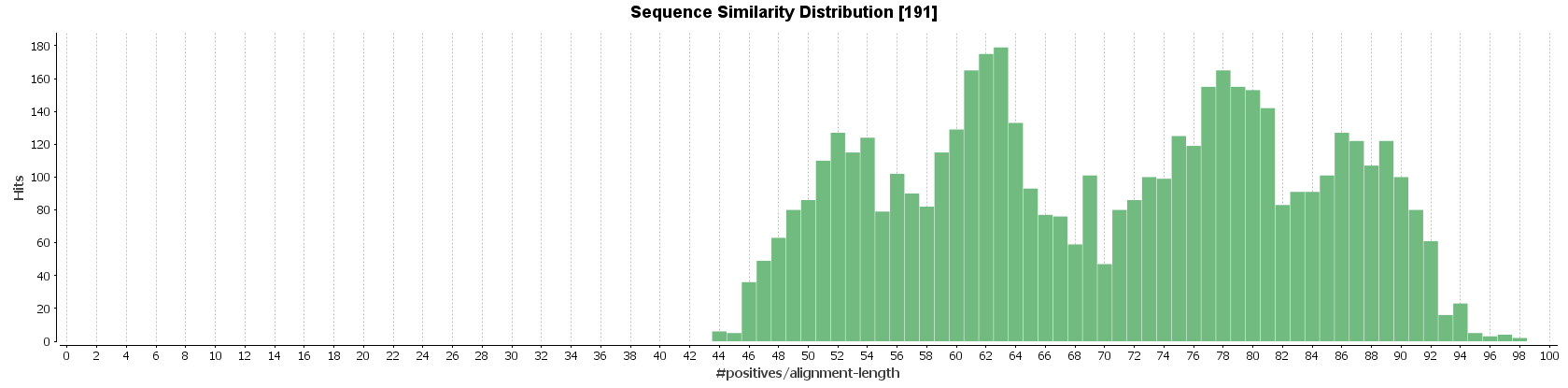


**A**

**B**

**C**

**Supplemntary Fig. S1.** Similarity distribution hits of *Brassica* with *Arabidopsis*. **(A)** *B. rapa* hits. **(B)** *B. oleracea* hits. **(C)** *B. napus* hits.


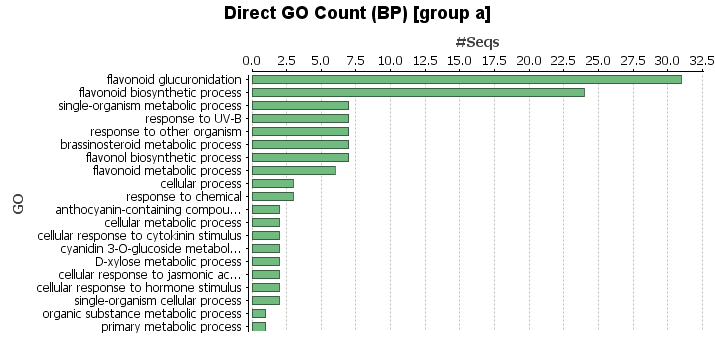


Group A biological process


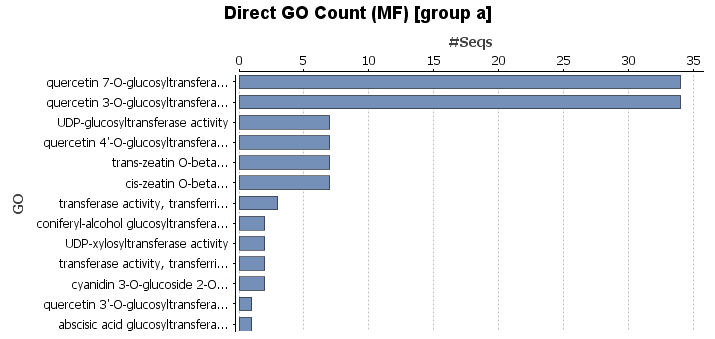


Group A molecular function


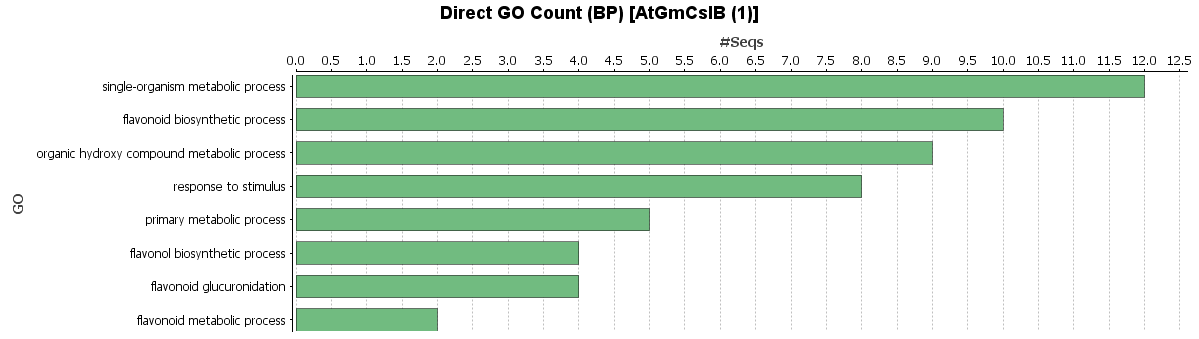


(Group B biological process)


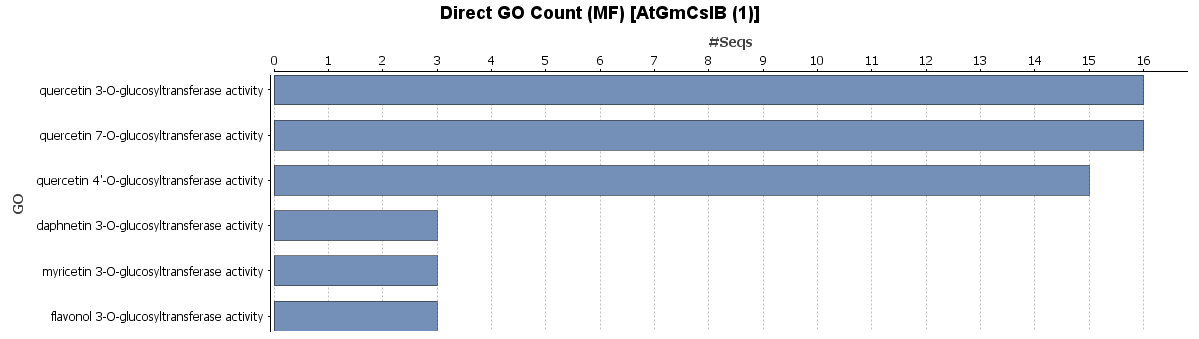


(Group B) molecular function


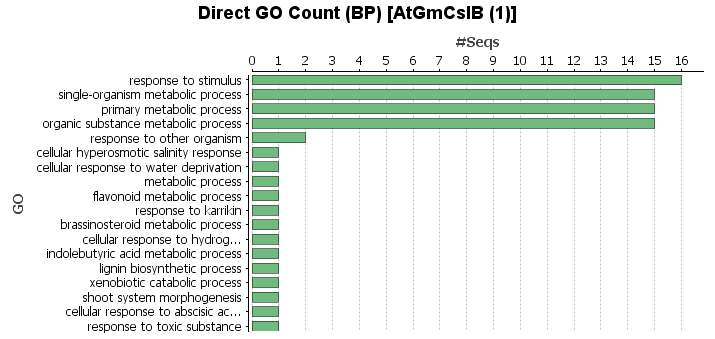


Group C biological process


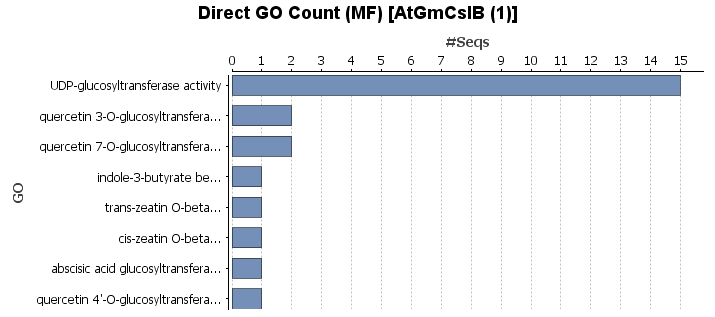


Group C molecular function


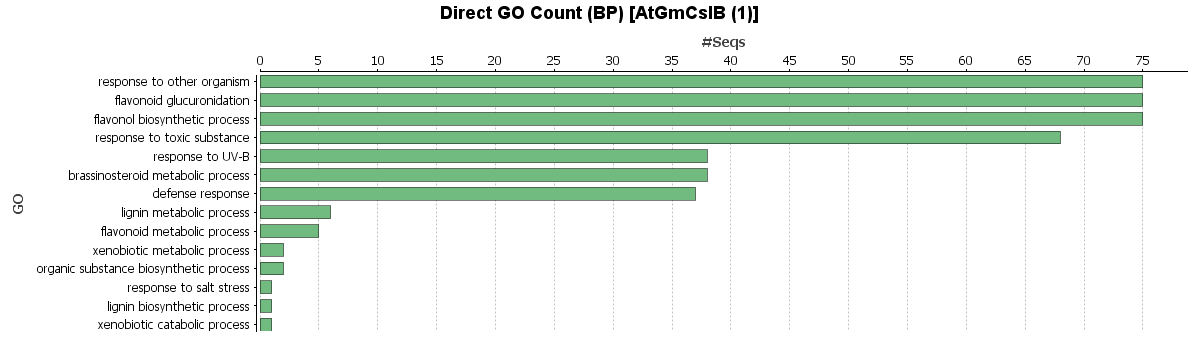


Group D biological process


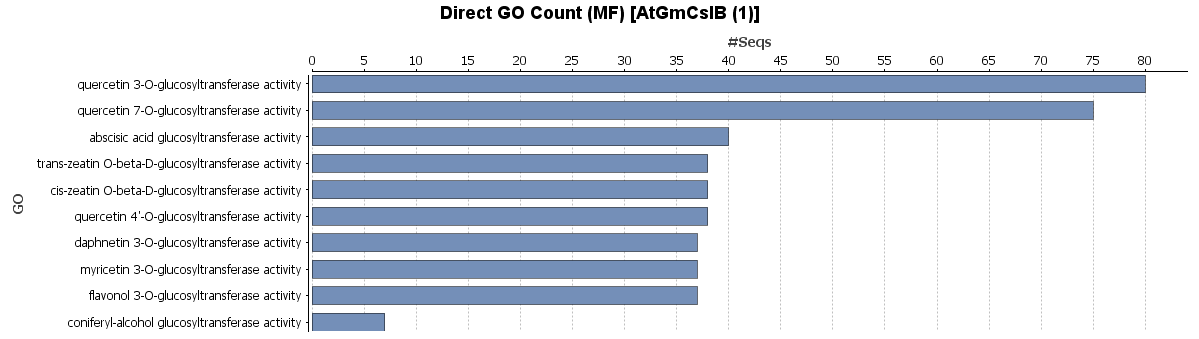


Group D molecular function


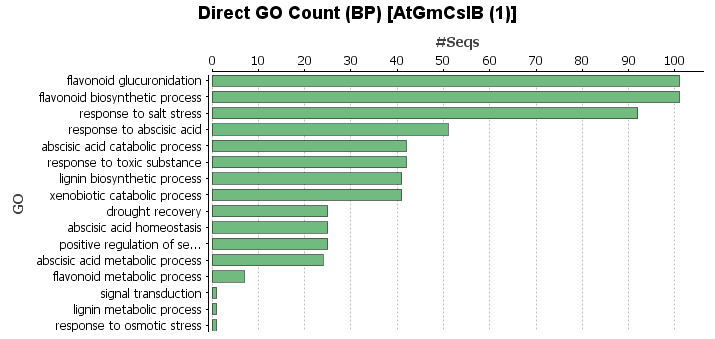


Group E biological process


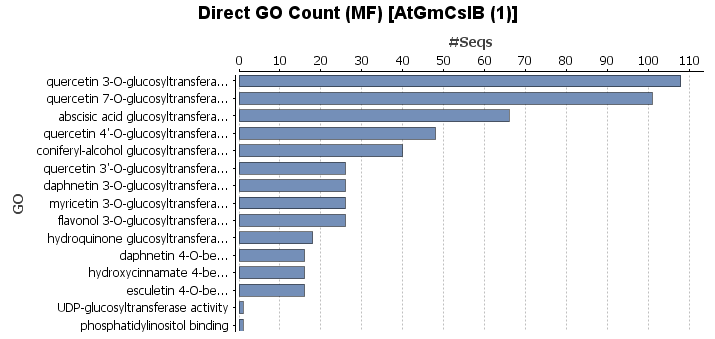


Group E molecular function


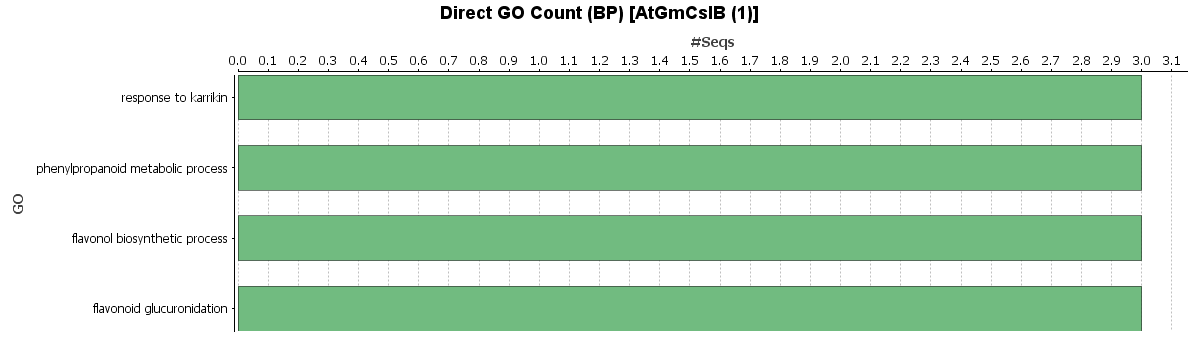


Group F biological process


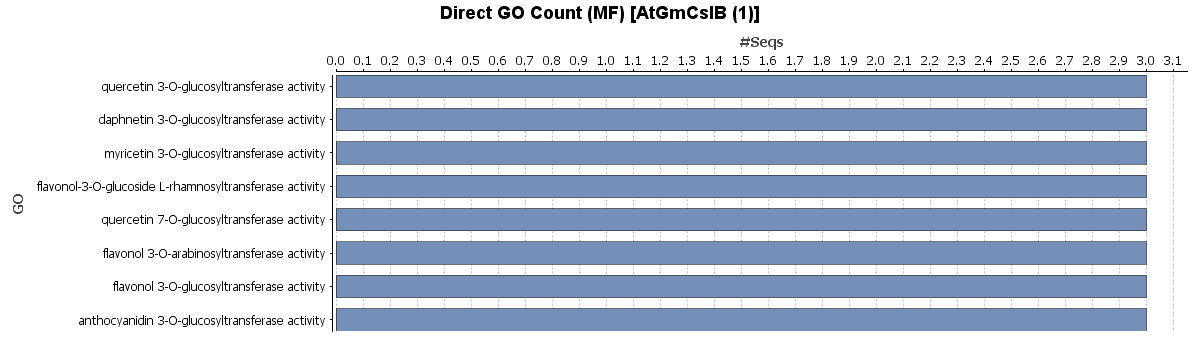


Group F molecular function


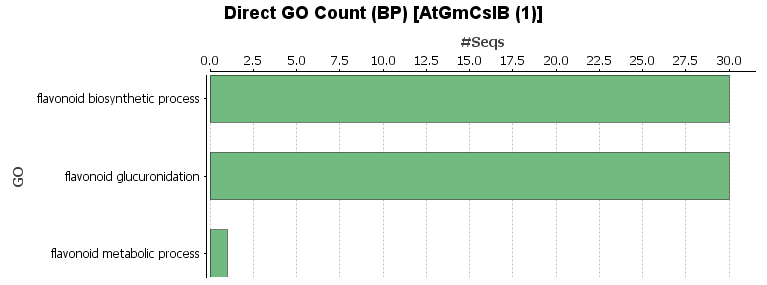


Group G biological process


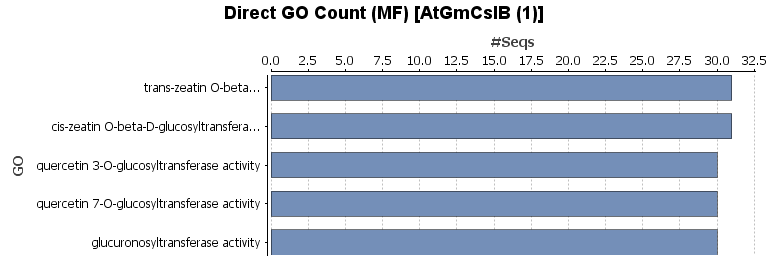


Group G molecular function


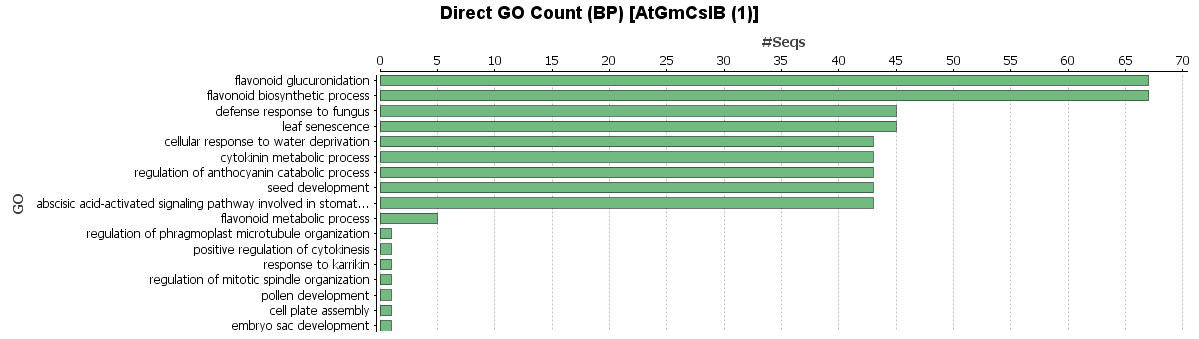


Group H biological process


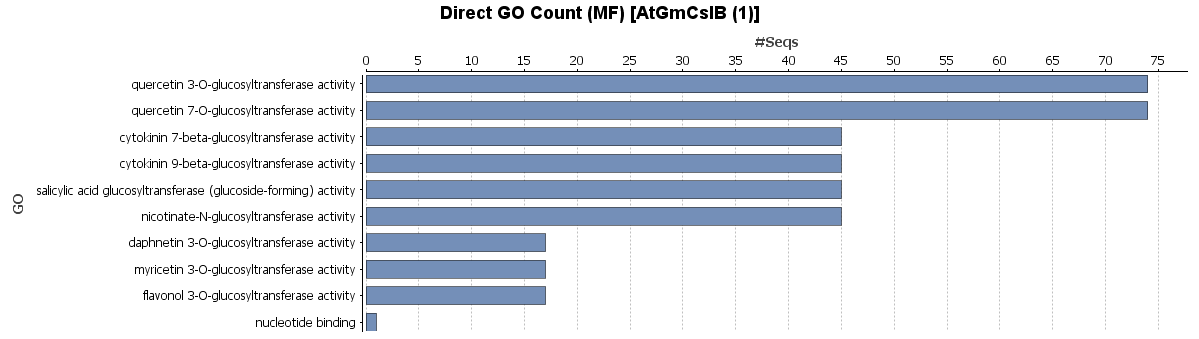


Group H molecular function


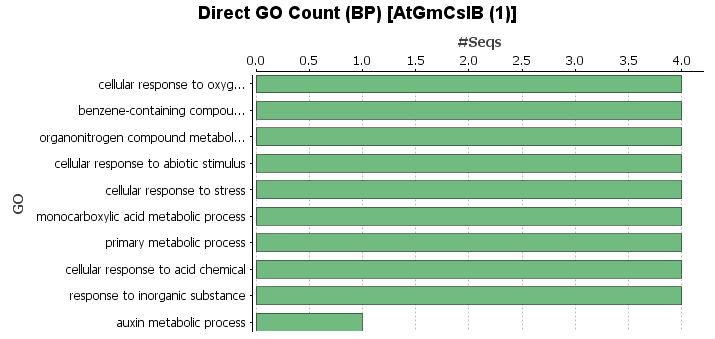


Group I biological process


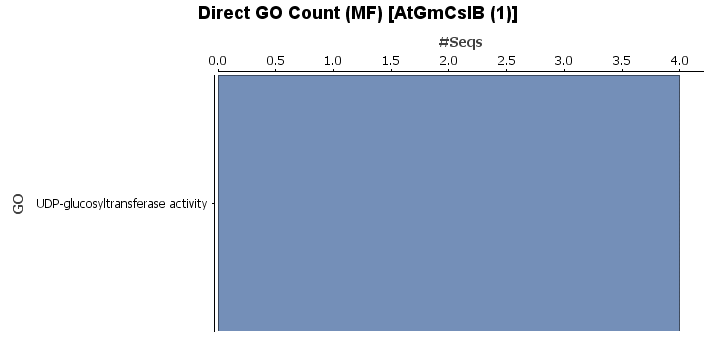


Group I molecular function


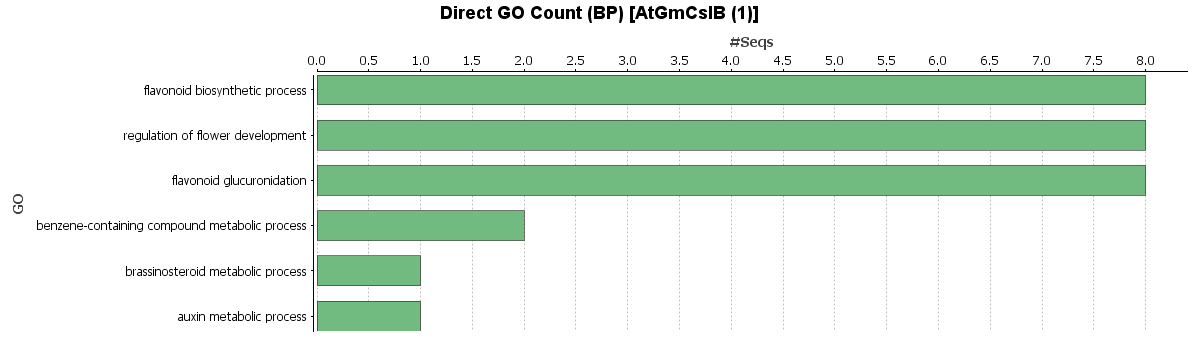


Group J biological process


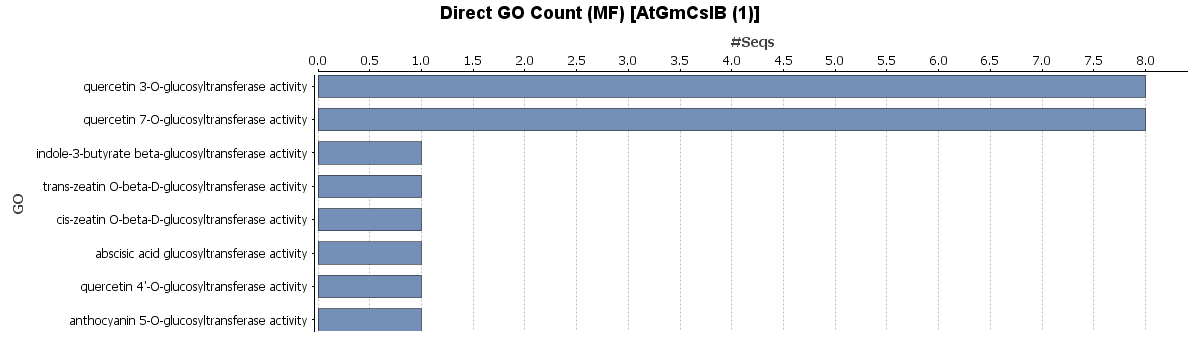


Group J molecular function


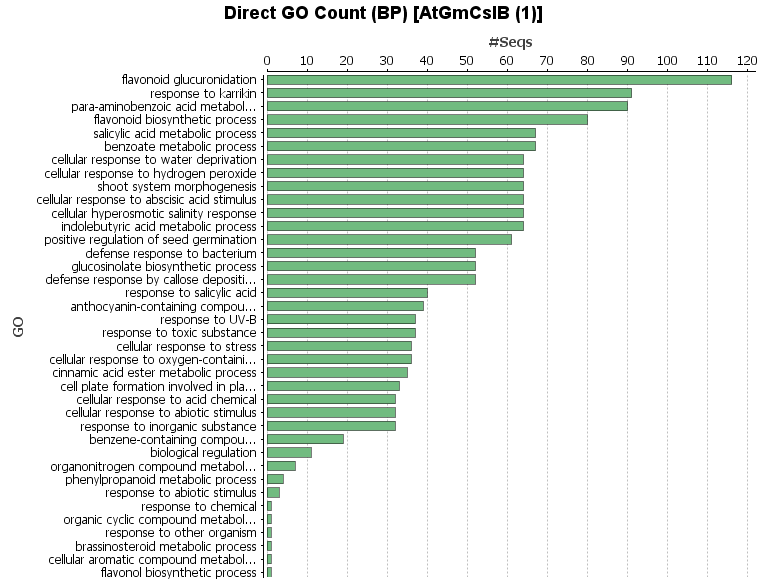


Group L biological process


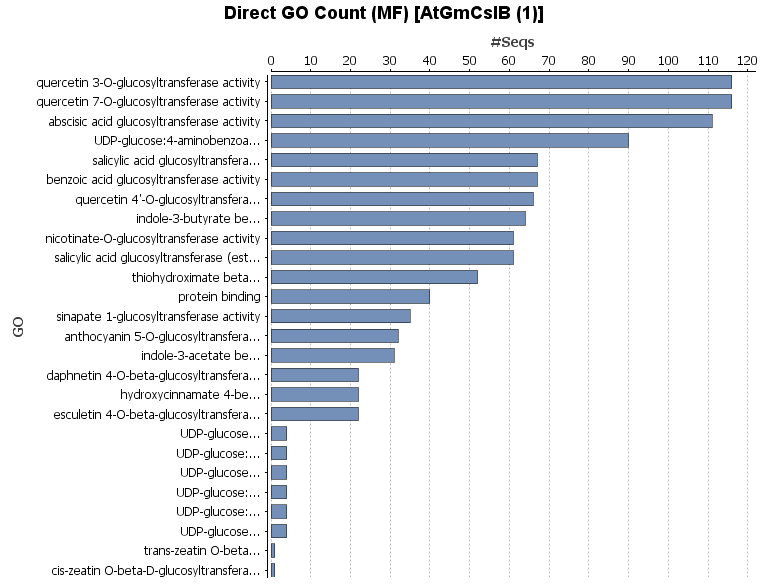


Group L molecular function


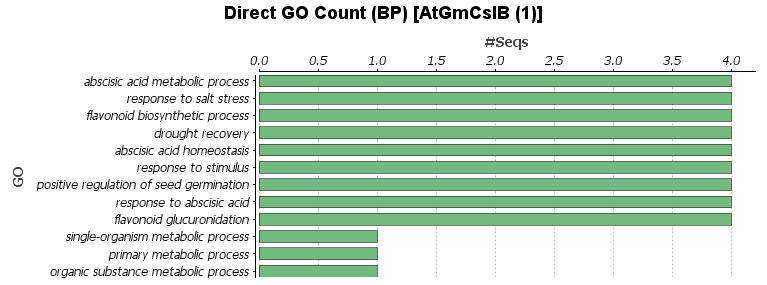


Group M biological process


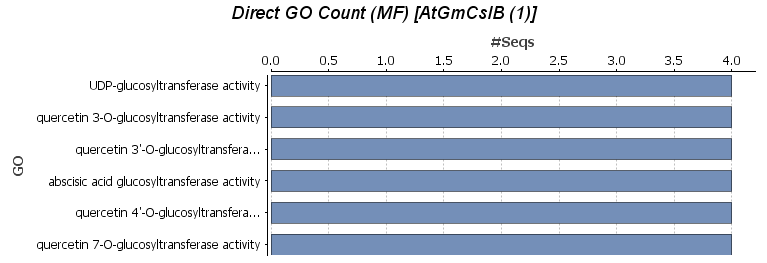


Group M molecular function

**Supplemntary Fig. S2** Phylogenetic group wise Go annotation analysis of all three Brassica species. X-axis is showing the number of genes, while the Y-axis showing annotated biological processes and molecular functions.


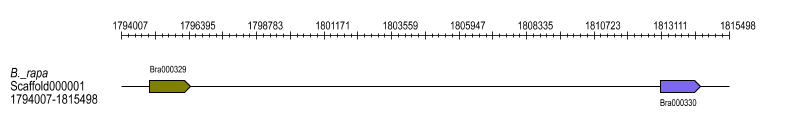


Cluster_672


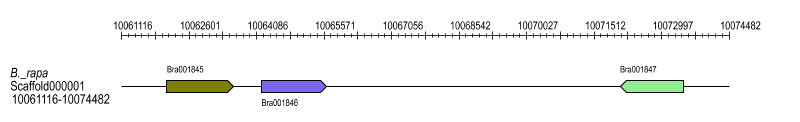


Cluster_245


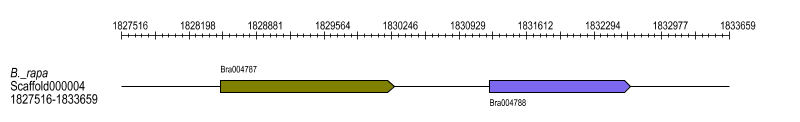


Cluster _1318


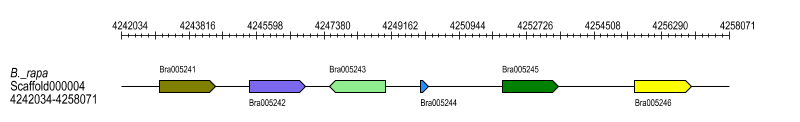


Cluster_17


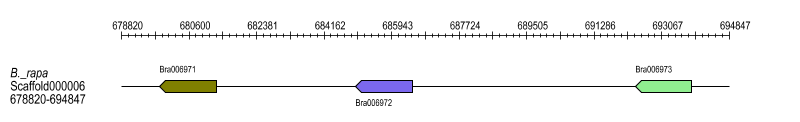


Cluster_171


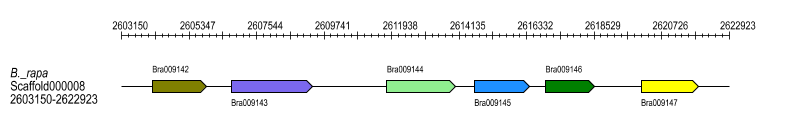


Cluster_13


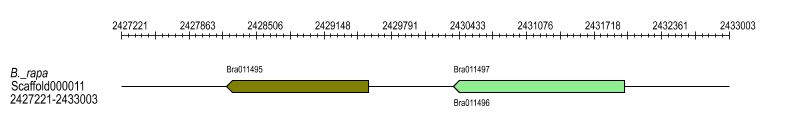


Cluster_422


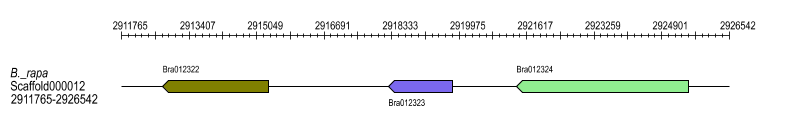


Cluster_339


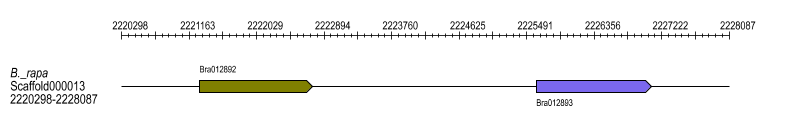


Cluster_712


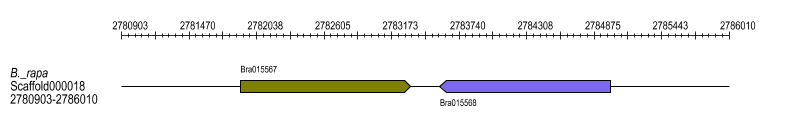


Cluster_1888


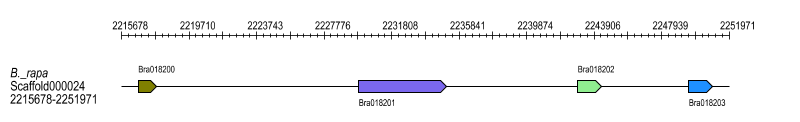


Cluster_76


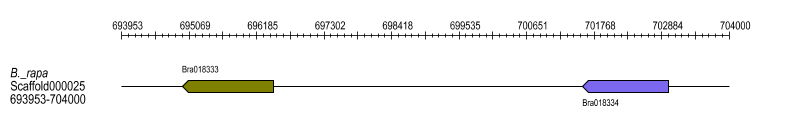


Cluster_1841


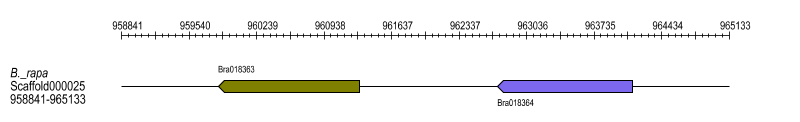


Cluster_1490


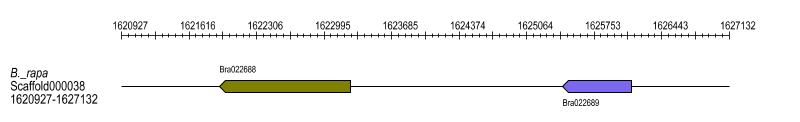


Cluster_1439


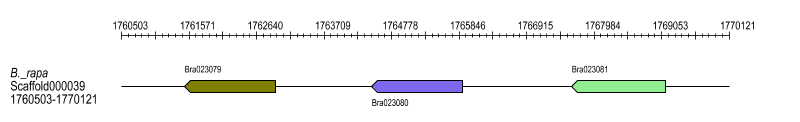


Cluster_375

Cluster_317


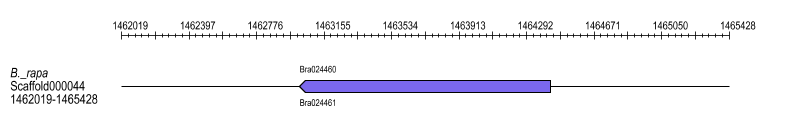


Cluster_904


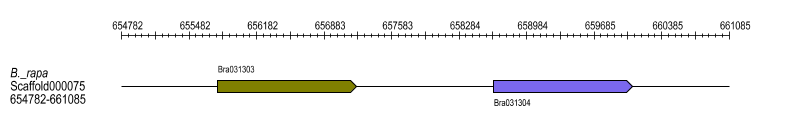


Cluster_1176


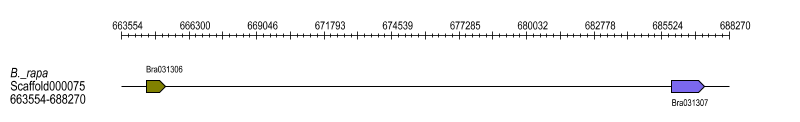


Cluster_1491


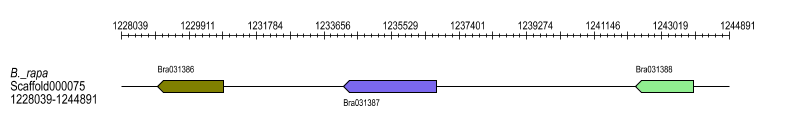


Cluster_317


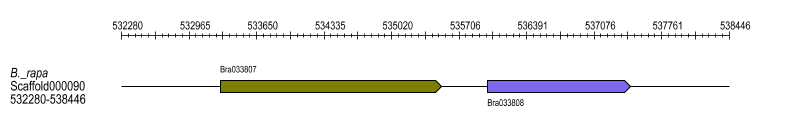


Cluster_1909


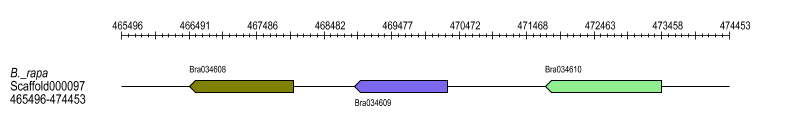


Cluster_283


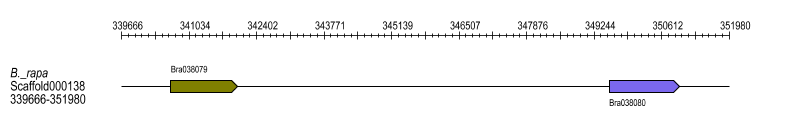
 Cluster_1610


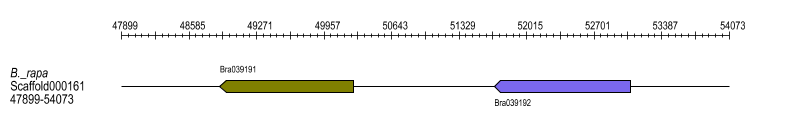


Cluster_595


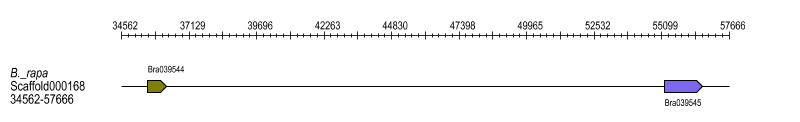


Cluster_1509


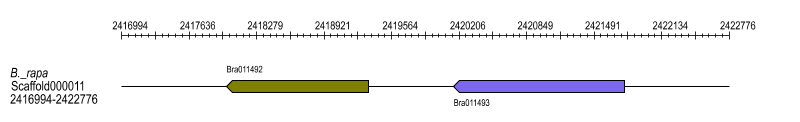


Cluster_1795


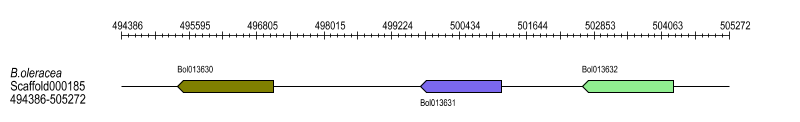


Cluster_146


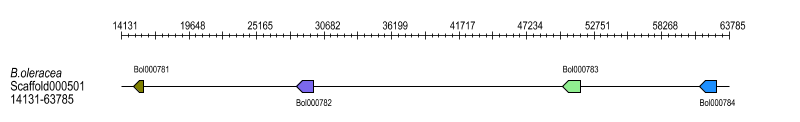


Cluster_42


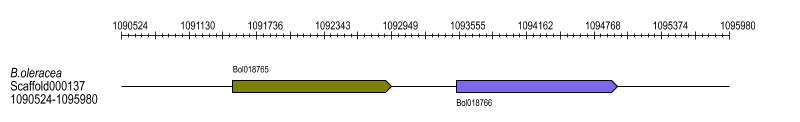


Cluster_1485


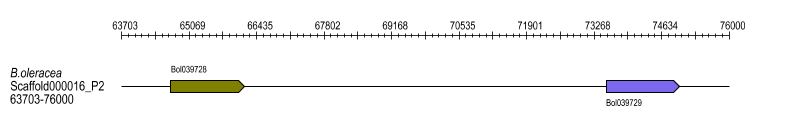


Cluster_482


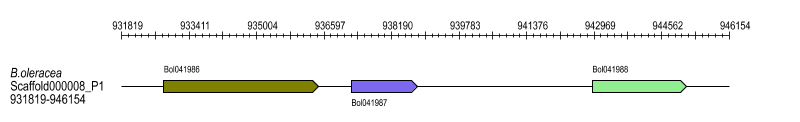


Cluster_272


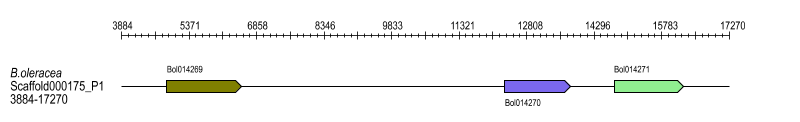


Cluster_166


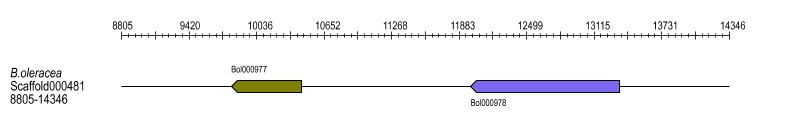


Cluster_1498


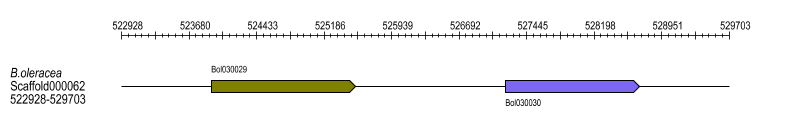


Cluster_453


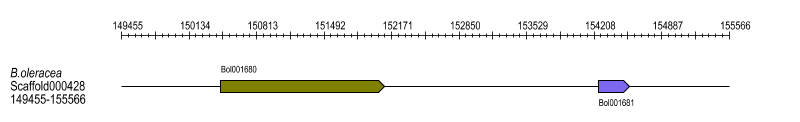


Cluster_1455


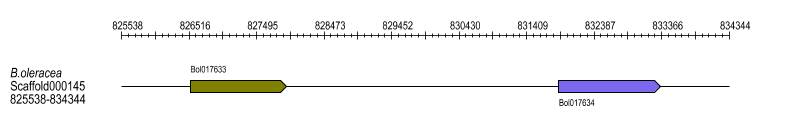


Cluster_1387


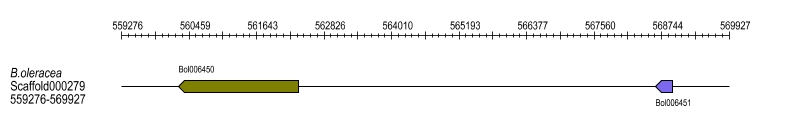


Cluster_1296


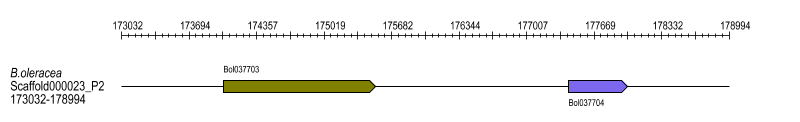


Cluster_491


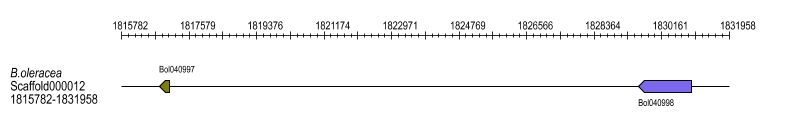


Cluster_354


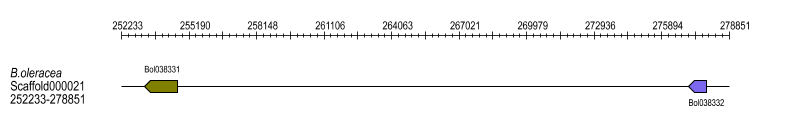


Cluster_334


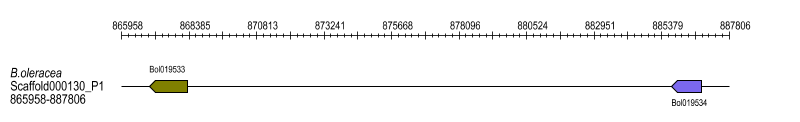


Cluster_1103


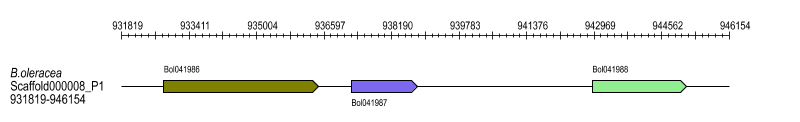


Cluster_272


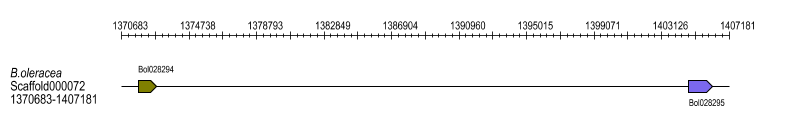


Cluster_539

**Supplemntary Fig. S3** Graphical representation of *B. rapa* and *B. oleracea* tandemly duplicated UGTs.


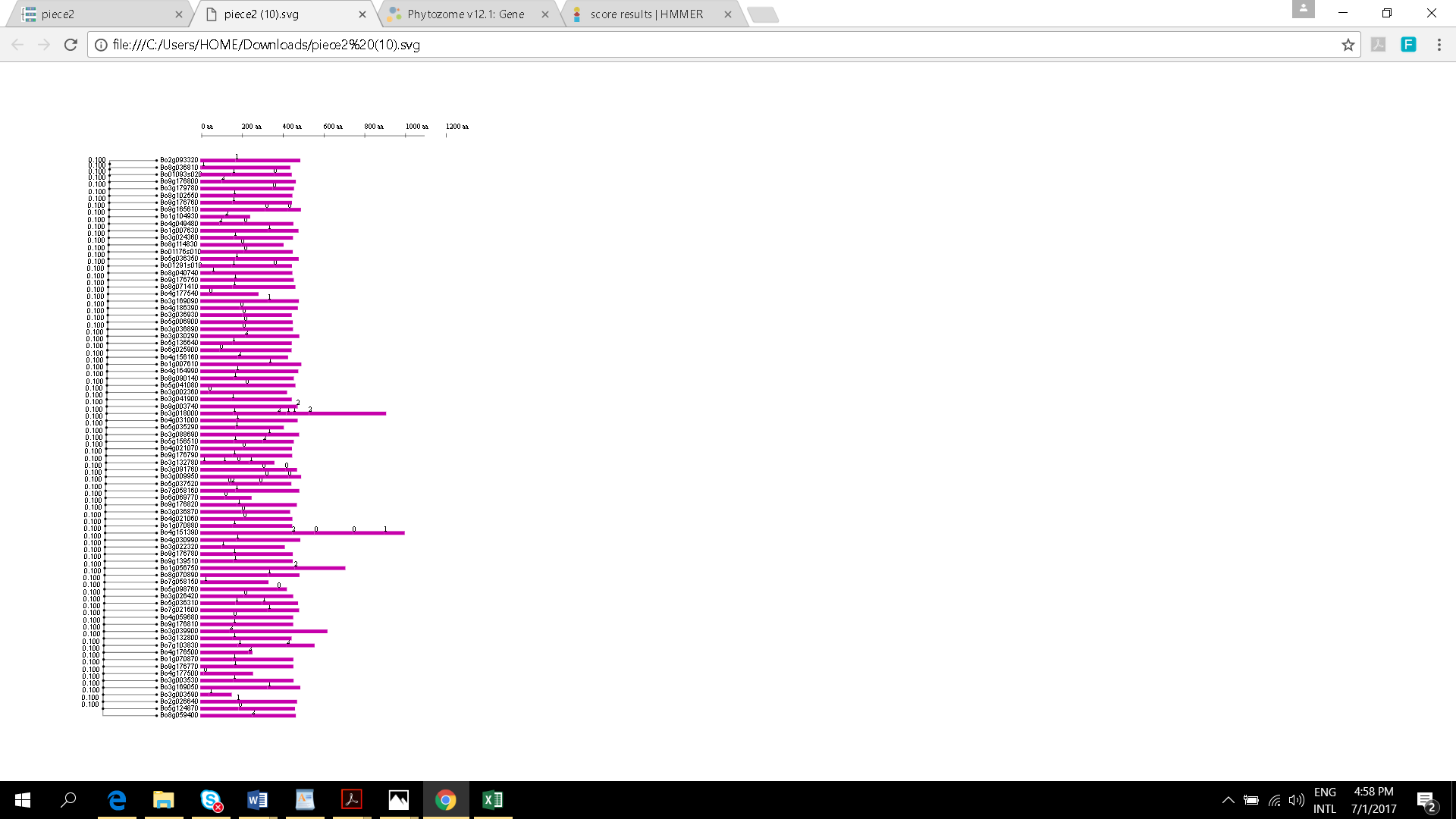


**A**


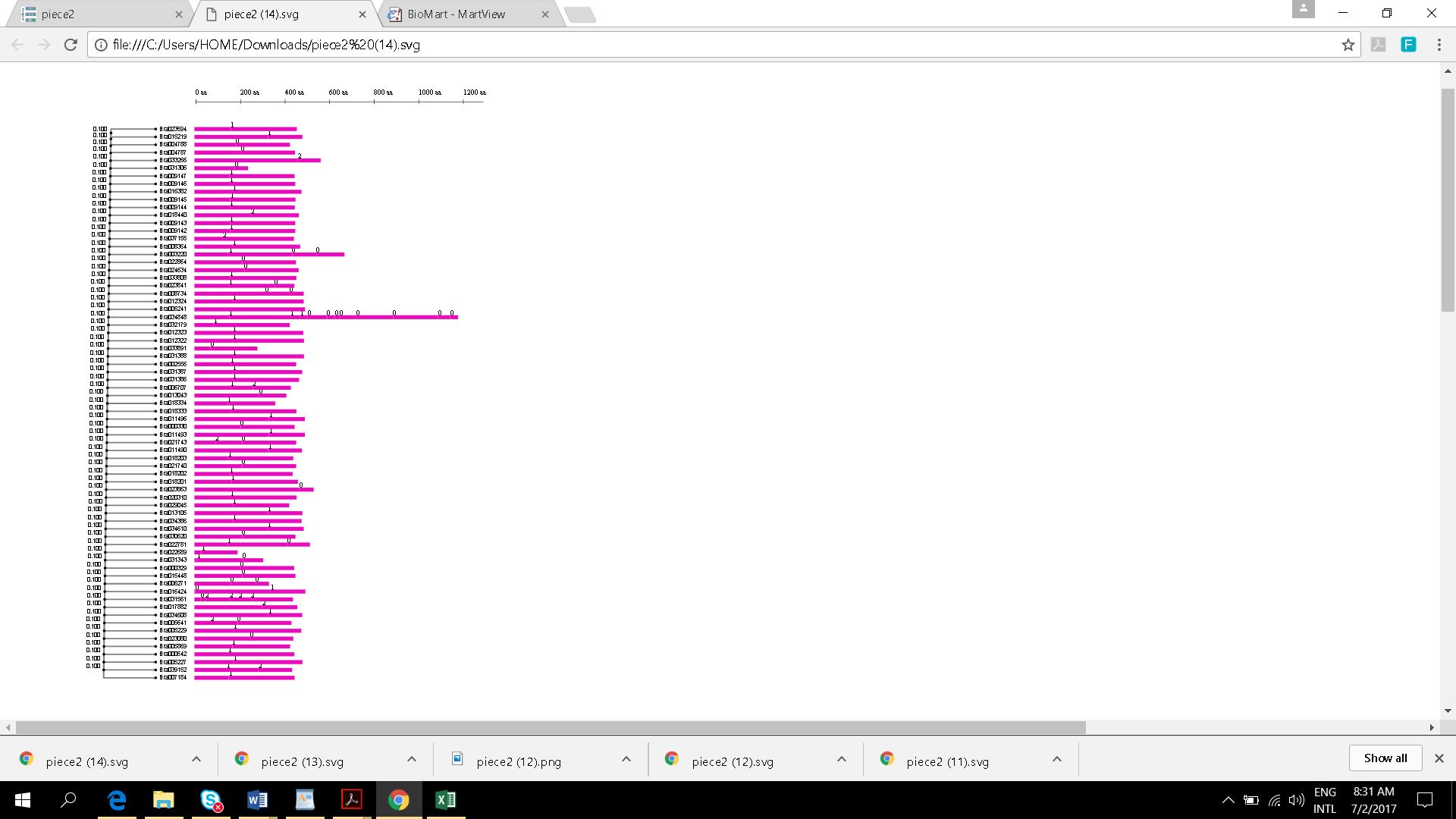


**B**


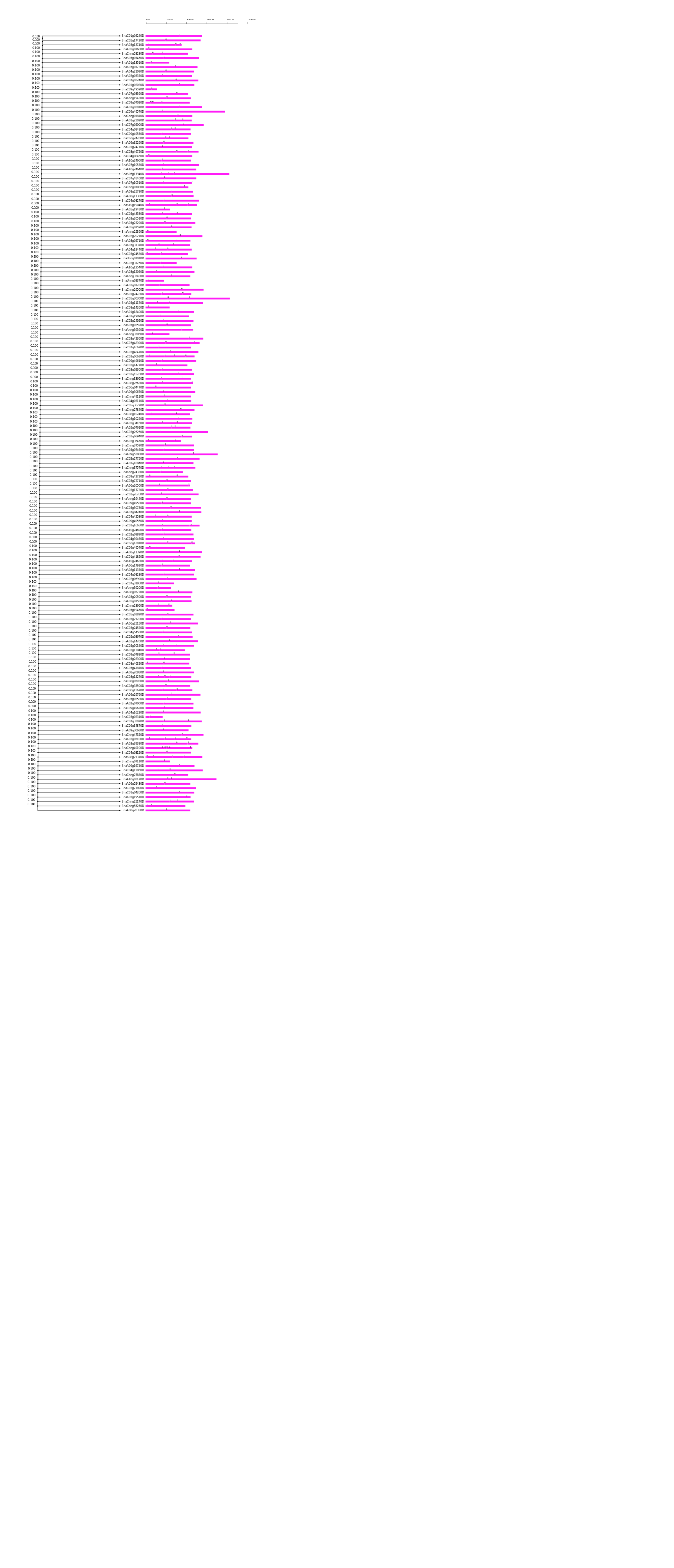


**C**

**Supplemntary Fig. S4** Intron organization among intron containing UGTs of all three *Brassica*. Red arrow is showing the conserved introns position between (150-200 amino acids) and phase1.


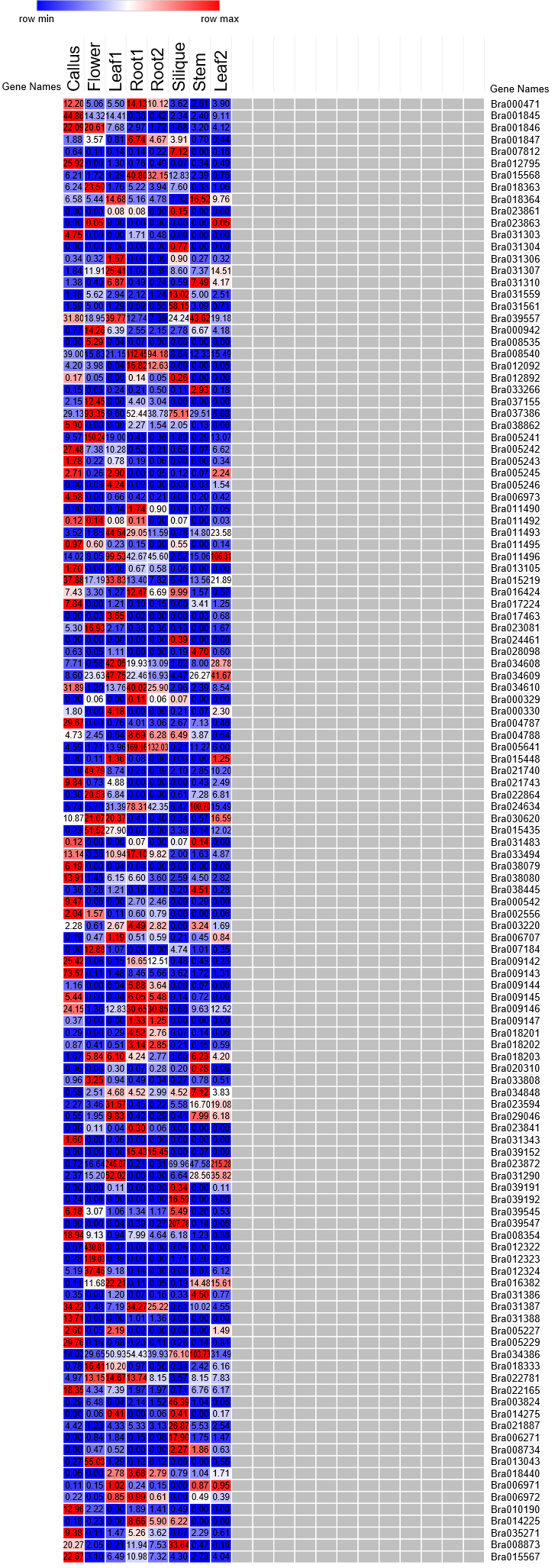

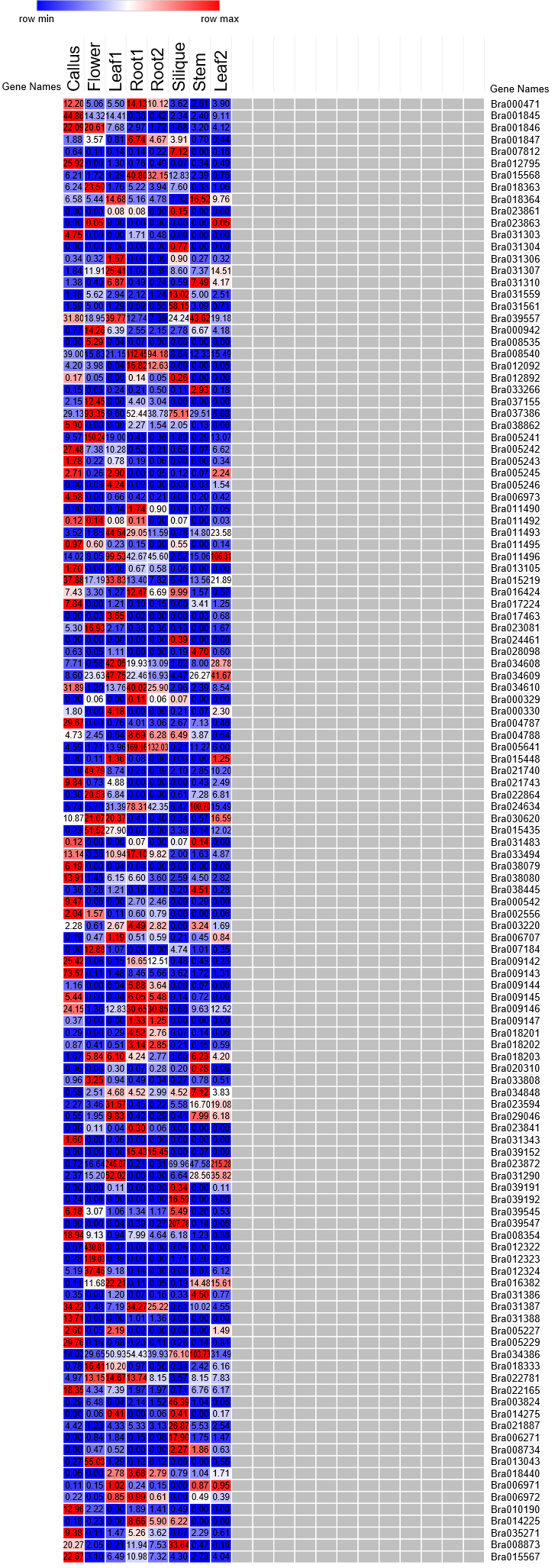


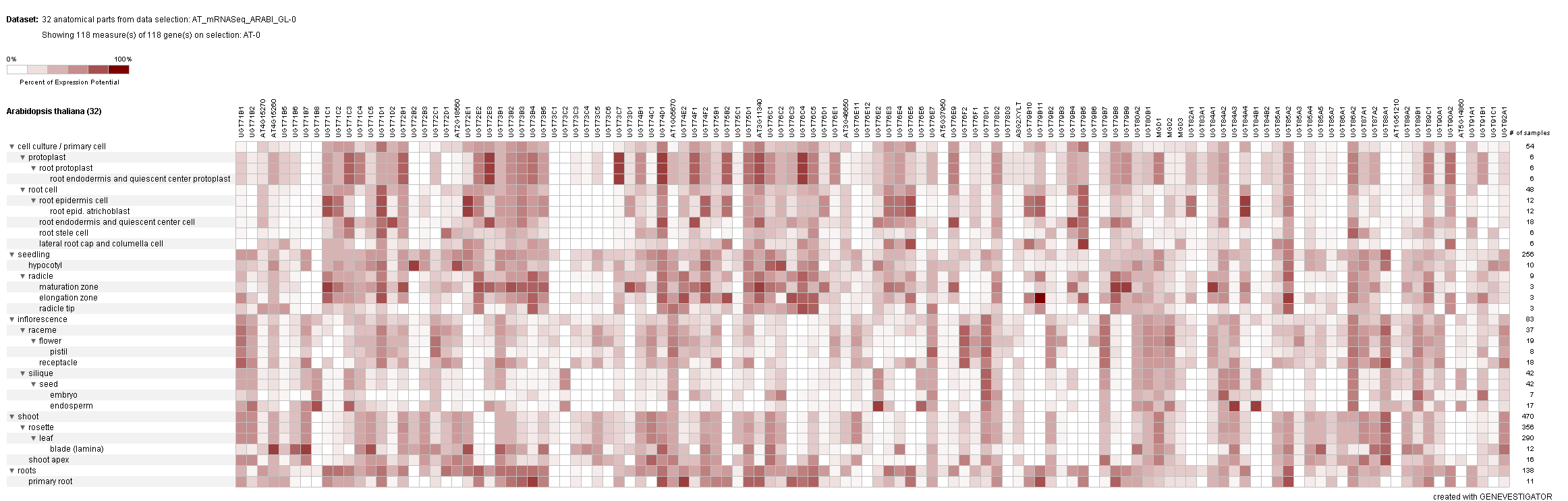

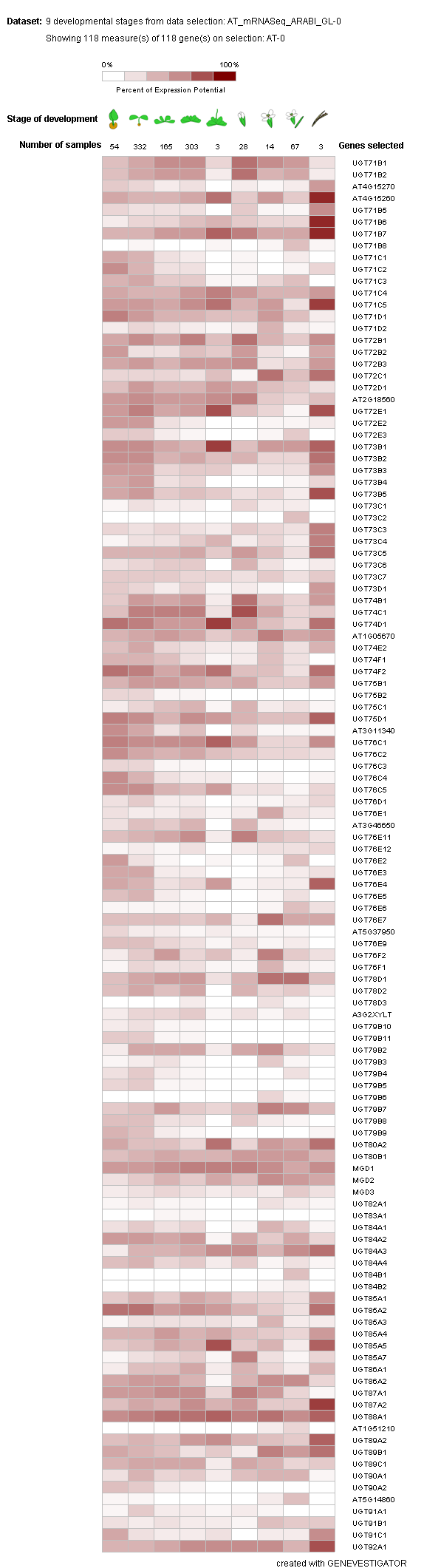


**A** **B**  **C**

**Supplemntary Fig. S5** Tissue specific expression of putative UGTs in *B.rapa* and *Arabidopsis*. **(A)** represenating expression of puative UGTs of *B. rapa* in callus, flower, root, leaf and silique. (B) representing expression of *Arabidopsis* UGTs in 32 various tissues. (C) representing expression of *Arabidopsis* UGTs at different developmental stages.


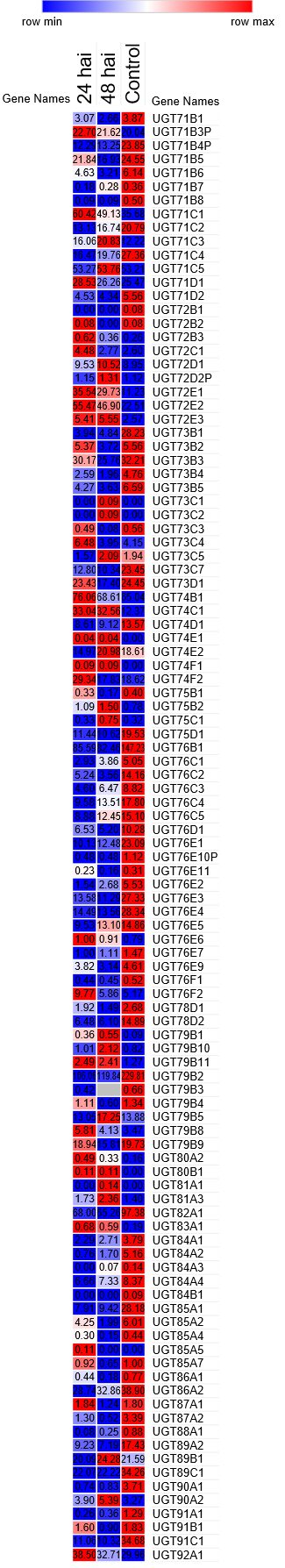

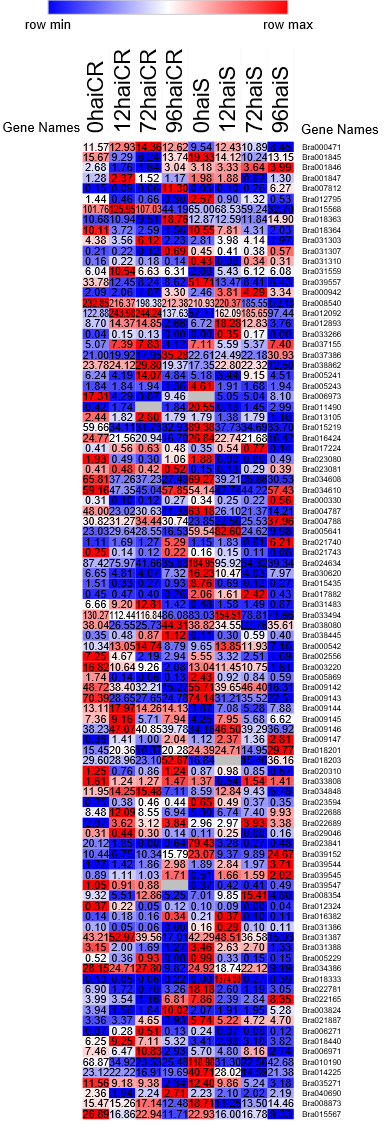


**A B**

**Supplemntary Fig. S6** RNA seq based expression heatmaps of *Arabidopsis* and *B. rapa* UGTs in response to *P. brassicae* infestation at various time stages. **(A)** representing expression of *Arabidopsis* UGTs at 24 hai and 48 hai stages. **(B)** representing expression of *B. rapa* putative UGTs in clubroot resistant and susceptible lines at various time stages.


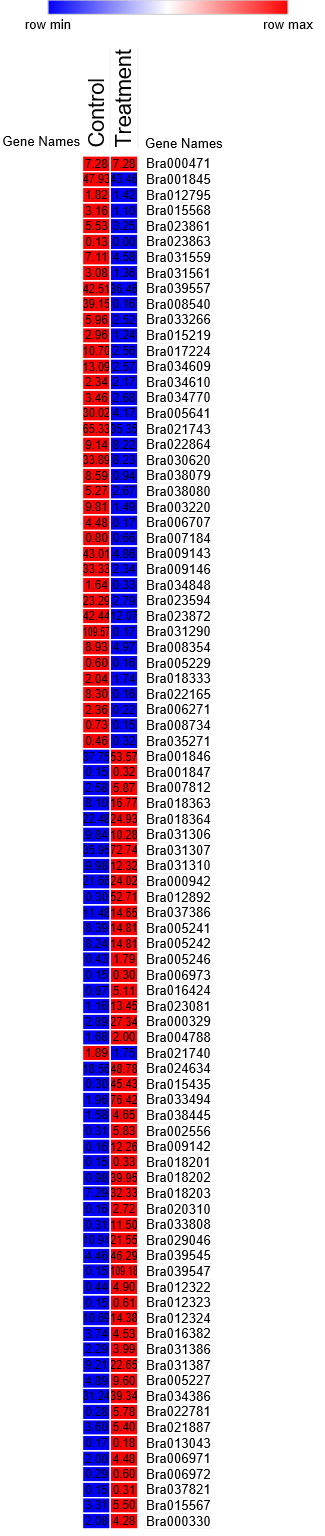


**Supplemntary Fig. S7** RNA Seq based expression of *B. rapa* putative UGTs in response to MeJa.
